# Lenvatinib plus anti-PD-1 antibody combination treatment activates CD8^+^ T cells through reduction of tumor-associated macrophage and activation of the interferon pathway

**DOI:** 10.1371/journal.pone.0212513

**Published:** 2019-02-27

**Authors:** Yu Kato, Kimiyo Tabata, Takayuki Kimura, Ayako Yachie-Kinoshita, Yoichi Ozawa, Kazuhiko Yamada, Junichi Ito, Sho Tachino, Yusaku Hori, Masahiro Matsuki, Yukiko Matsuoka, Samik Ghosh, Hiroaki Kitano, Kenichi Nomoto, Junji Matsui, Yasuhiro Funahashi

**Affiliations:** 1 Tsukuba Research Laboratories, Eisai Co., Ltd., Tsukuba, Ibaraki, Japan; 2 The Systems Biology Institute, Shinagawa, Tokyo, Japan; 3 Oncology Business Group, Eisai Inc., Woodcliff Lake, New Jersey, United States of America; Istituto Superiore di Sanità, ITALY

## Abstract

Lenvatinib is a multiple receptor tyrosine kinase inhibitor targeting mainly vascular endothelial growth factor (VEGF) and fibroblast growth factor (FGF) receptors. We investigated the immunomodulatory activities of lenvatinib in the tumor microenvironment and its mechanisms of enhanced antitumor activity when combined with a programmed cell death-1 (PD-1) blockade. Antitumor activity was examined in immunodeficient and immunocompetent mouse tumor models. Single-cell analysis, flow cytometric analysis, and immunohistochemistry were used to analyze immune cell populations and their activation. Gene co-expression network analysis and pathway analysis using RNA sequencing data were used to identify lenvatinib-driven combined activity with anti-PD-1 antibody (anti-PD-1). Lenvatinib showed potent antitumor activity in the immunocompetent tumor microenvironment compared with the immunodeficient tumor microenvironment. Antitumor activity of lenvatinib plus anti-PD-1 was greater than that of either single treatment. Flow cytometric analysis revealed that lenvatinib reduced tumor-associated macrophages (TAMs) and increased the percentage of activated CD8^+^ T cells secreting interferon (IFN)-γ^+^ and granzyme B (GzmB). Combination treatment further increased the percentage of T cells, especially CD8^+^ T cells, among CD45^+^ cells and increased IFN-γ^+^ and GzmB^+^ CD8^+^ T cells. Transcriptome analyses of tumors resected from treated mice showed that genes specifically regulated by the combination were significantly enriched for type-I IFN signaling. Pretreatment with lenvatinib followed by anti-PD-1 treatment induced significant antitumor activity compared with anti-PD-1 treatment alone. Our findings show that lenvatinib modulates cancer immunity in the tumor microenvironment by reducing TAMs and, when combined with PD-1 blockade, shows enhanced antitumor activity via the IFN signaling pathway. These findings provide a scientific rationale for combination therapy of lenvatinib with PD-1 blockade to improve cancer immunotherapy.

## Introduction

Immune escape of cancer cells is a major mechanism of cancer malignancy, mainly due to exhaustion of CD8^+^ T cells that recognize tumor antigens [1]. The PD-1 signaling pathway is a key regulator of CD8^+^ T cell exhaustion [2]. PD-1 blockade was first approved for use in malignant melanoma patients and then in several other tumor types, such as renal cell carcinoma and non-small cell lung cancer [3–6], and further clinical trials are ongoing. However, overall response rates to single treatment with PD-1 blockade are approximately 20%–30% in patients with solid cancers [3, 4, 7]. Therefore, although PD-1 blockade therapy can be effective in many types of cancer, further improvements are required, and combination treatments with other therapies are being investigated in clinical trials.

Lenvatinib mesilate (lenvatinib) selectively inhibits VEGFR1–3 and other proangiogenic and oncogenic pathway-related receptor tyrosine kinases (RTKs), including FGFR1–4, platelet-derived growth factor receptor α (PDGFRα), KIT, and RET [8–10]. Lenvatinib is currently used as monotherapy in patients with radio-iodine-refractory differentiated thyroid cancer (RAI-DTC) [11] and in combination with everolimus to treat advanced renal cell carcinoma after VEGF therapy in the United States and the European Union [12]. Recently, lenvatinib was approved for first-line treatment of unresectable hepatocellular carcinoma (HCC) [13] in the United States, the European Union, Japan, China and other countries. Exploratory biomarker analysis of lenvatinib in a Phase 3 study of patients with RAI-DTC revealed that lenvatinib increased VEGF and FGF23 levels in serum and that the increased FGF23 levels were associated with progression-free survival, thus demonstrating the target engagement of lenvatinib to inhibit VEGFR and FGFR signaling [14].

In the present study, we investigated the antitumor and immunomodulatory activities of lenvatinib in syngeneic mouse tumor models. We also explored the mechanism of action of combination treatment with lenvatinib plus PD-1 blockade by using flow cytometry, immunohistochemistry, and transcriptome analyses of tumor tissues treated with lenvatinib, PD-1 blockade, or lenvatinib plus PD-1 blockade. Our data suggest that lenvatinib exhibits immunomodulatory activity by decreasing TAM numbers and that combined treatment with PD-1 blockade induces potent antitumor activities via the activation of IFN signaling pathways in cancer immune microenvironments.

## Materials and methods

### In vivo antitumor activity study

CAnN.Cg-Foxn1nu/CrlCrlj (Balb/c^nu/nu^) and BALB/cAnNCrlCrlj (Balb/c^wt/wt^) mice were obtained from Charles River Laboratories Japan, and C57BL/6JJcl mice came from CLEA Japan. BNL 1ME A.7R.1, a murine hepatocellular carcinoma cell line, and CT26, a murine colon carcinoma cell line, were purchased from ATCC. B16-F10, a murine melanoma cell line, was kindly provided by Dr. Isaiah J. Fidler (MD Anderson Cancer Center, Houston, Texas, USA). Lenvatinib was synthesized at Eisai (Ibaraki, Japan) and diluted with distilled water containing 3 mM HCl. Cells (2.0–5.0 × 10^6^) in 100 μL of PBS were injected subcutaneously into the mice. When the mean tumor volume reached 30–170 mm^3^, the treatments were initiated. All animals received lenvatinib (3 or 10 mg/kg orally) once daily for the duration of time indicated by the black arrow in the figures. Anti-mouse PD-1 antibody (clone; RMP1-14), anti-mouse CD8 antibody (clone; YTS169.4), anti-mouse IFN-γ antibody (clone; R4-6A2), anti-mouse CSF1R antibody (clone; AFS98), and isotype control antibodies for each antibody were purchased from Bio X Cell (West Lebanon, NH, USA). All antibodies were diluted with PBS and mice were dosed as follows: anti-PD-1 antibody, 200 μg/mouse, once every three days or twice weekly; anti-CD8 antibody, 200 μg/mouse, two days before drug treatment and twice weekly thereafter; anti-IFN-γ antibody, 300 μg/mouse, one day before treatment and twice weekly thereafter; and anti-CSF1R antibody, 300 μg/mouse, one day before drug treatment and once every two days thereafter.

The tumor volume was measured twice weekly. Tumor size was calculated as follows: Tumor volume (mm^3^) = Long axis (mm) × Short axis (mm) × Short axis (mm) / 2. The percentage of ΔT/C (% of control for Δgrowth) was calculated using the following formula: (ΔT/ΔC) × 100%, where ΔT and ΔC are the changes in tumor volume (TV) (Δgrowth) for the drug-treated and non-treated control groups, respectively. Animal care and experimental procedures were performed in an animal facility accredited by the Health Science Center for Accreditation of Laboratory Animal Care and Use of the Japan Health Sciences Foundation. Our ethics committee is “Institutional Animal Care and Use Committee”. All protocols were approved by the Institutional Animal Care and Use Committee and carried out in accordance with the Animal Experimentation Regulations of Eisai Co., Ltd.

### Flow cytometric analysis

Tumor tissues were collected from each mouse on Day 8 or 9 after treatment, dissociated into single cells by using a Tumor Dissociation Kit and a gentleMACS Dissociator (Miltenyi Biotec, Bergisch Gladbach, Germany). In cell mixtures, leukocytes positive for CD45 were isolated with mouse TIL (CD45) microbeads (Miltenyi Biotec) by using an OctoMACS Separator (Miltenyi Biotec). After washing and filtration, cells were blocked with Mouse BD Fc Block (BD Biosciences, San Jose, CA, USA), and stained with the antibodies listed in S1 Table and with DAPI (4′,6-diamidino-2-phenylindole, Dojindo, Kumamoto, Japan). The gating strategy is shown in S1 Fig. To detect IFN-γ-producing activated CD8^+^ T cells, we incubated CD45^+^ cells with RPMI1640 containing with 10% FBS, PMA/Ionomycin (Sigma, St. Louis, Missouri, USA), and BD GolgiPlug (BD Biosciences) for 4 h at 37 °C, and then stained them with antibodies against CD45, CD3 (Thermo Fisher Scientific, Waltham, MA, USA), CD4, and CD8 (BD Biosciences). The cells were then fixed and permeabilized with BD Cytofix/Cytoperm (BD Biosciences) and then stained with antibody against IFN-γ or GzmB (BD Biosciences). The cells were sorted with a BD FACS AriaII SORP or LSRFortessa X-20 (BD Biosciences) and the data were analyzed by using FlowJo v10 (BD Biosciences) or Cytobank (Cytobank, Inc., Santa Clara, CA, USA).

### Immunohistochemistry

Tumor tissues were embedded in Tissue-Tek O.C.T. compound (Sakura Finetek, Nagano, Japan) and frozen. Section slices were placed on glass slides and fixed with cold methanol for 10 min, dried, and then blocked with Block Ace buffer (DS Pharma Biomedical, Osaka, Japan) for 20 min at room temperature. Rabbit anti-mouse CD11b antibody, rat anti-F4/80 antibody (Abcam, Cambridge, UK), and anti-mouse CD31 antibody diluted with Block Ace buffer were added to the slides, which were then incubated for 60 min at room temperature. The slides were then washed three times with PBS. Anti-rat IgG-Alexa488, and anti-rabbit IgG-Alexa594, diluted with Block Ace buffer, were then added to the slides, which were again incubated for 60 min at room temperature before being washed three times with PBS. Image scanning was conducted on a Leica TCS SP8 (Leica Microsystems, Wetzlar, Germany) or NanoZoomer (Hamamatsu Photonics, Shizuoka, Japan); the images were analyzed on a LAS AF (Leica Microsystems) or HALO (Indica Labs, Corrales, NM, USA).

### Single-cell analysis

Tumor tissues were collected from mice bearing BNL 1ME A.7R.1 tumors after 1 week of treatment with lenvatinib. After dissociation with a tumor dissociation kit (Miltenyi Biotec), CD45^+^ cells were isolated by using a magnetic activated cell sorting (MACS) system with CD45 microbeads (Miltenyi Biotec). Single-cell RNA sequencing libraries of extracted CD45^+^ cells were prepared by using a ddSEQ Single-Cell Isolator (Bio-Rad Laboratories, Hercules, CA, USA) and a SureCell WTA 3’ Library Prep Kit (Illumina, San Diego, CA, USA). Data preprocessing, such as unique molecule identifier (UMI) counting, was performed on the BaseSpace according to the manufacturer’s instructions. Cells that passed quality control with total UMIs of between 400 and 10,000 were selected for further analysis. To standardize gene expression across cells, UMIs were converted to UMIs per million (UPM) by dividing the UMI of each gene by the total UMIs of the cell and then multiplying by a scale factor of 1 million. Finally, the UPM was further transformed to the logarithmic scale [log_2_(UPM+1)]. To visualize variations in immune cells and estimate the effect of lenvatinib treatment on immune cells, t-distributed stochastic neighbor embedding (t-SNE) [15] was performed using the gene expression of 750 immune-related genes [16].

### RNA sequencing (RNA-Seq)

The CT26 tumor tissues were extracted on day 9 after each treatment, and using RNA from the tumor tissues, sequencing libraries were generated by using an Illumina TruSeq mRNA Library Kit according to the manufacturer’s instructions. Paired-end sequencing of 2 × 150 bp was conducted using an Illumina NextSeq 500 according to the manufacturer’s instructions. FASTQ files for each sample converted from the BCL file were entered into FASTQC (http://www.bioinformatics.babraham.ac.uk/projects/fastqc/) for quality assessments. Sequencing data were processed by using Trimmomatic (v0.36) [17] to remove all Illumina adaptor sequences and bases with low quality scores. Filtered reads were mapped to the mouse genome (Mm10 and Ensembl 84) by using STAR (v2.5.0c) [18]. Gene expression levels in transcripts per million (TPM) for all samples were estimated using RSEM (v1.2.31) [19].

Data analysis was conducted by using Strand NGS (Agilent Technology, Santa Clara, CA, USA) and Ingenuity Pathway Analysis (IPA, QIAGEN, Hilden, Germany).

### Weighted gene co-expression network analysis

Weighted gene co-expression network analysis (WGCNA) [20] was applied to identify gene modules in the RNA-Seq transcriptome data of the CT26 tumor tissues by using the R Bioconductor package. WGCNA clusters genes into color-name assigned modules using a topological overlap that measures network connectedness from an adjacency matrix of genes. A soft-thresholding power was determined to approximate scale-free topology of the network. The gene modules were identified by using a dynamic branch-cutting algorithm [21] where the dissimilarity correlation threshold and the minimum number of genes in each cluster were set as 0.05 and 30, where the modules reflecting potentiating effects emerged.

### Gene enrichment analysis

Pathway enrichment analysis for a given gene list was performed by simple overlap statistic (hypergeometric) analysis [22]. The biologically meaningful gene ontology terms and pathways were collected from multiple databases including the Gene Ontology consortium (GO; http://geneontology.org), Pathway Interaction Database (PID; http://pid.nci.nih.gov), Reactome (http://www.reactome.org), Wikipathways (www.wikipathways.org/), and NetPath (www.netpath.org/).

### Quantitative real-time PCR (qRT-PCR)

The CT26 tumor tissues were extracted on Day 8 after antibodies treatment, and using RNA from the tumor tissue samples, cDNA was made using high-capacity cDNA Reverse Transcription Kit (Thermo Fisher Scientific). The cDNA diluted with TaqMan Fast Advanced Master Mix (Thermo Fisher Scientific) was applied in custom designed TaqMan array microfluidic cards (Thermo Fisher Scientific). Gene expression was measured by QuantStudio7 (Thermo Fisher Scientific). The data analysis was conducted by using Expression Suite Software v1.1.

### Statistical analysis

Differences between vehicle- and mono- or combination therapy-treated groups were analyzed by using unpaired t tests. A value of *P* < 0.05 (two sided) was considered statistically significant. Missing tumor volume data were imputed using the last observation carry forward (LOCF). Statistical analyses were performed by using Prism (v7.02, GraphPad Software, San Diego, California, USA).

## Results

### Immunomodulating and antitumor activity of lenvatinib under immunocompetent conditions

**Fig 1:**
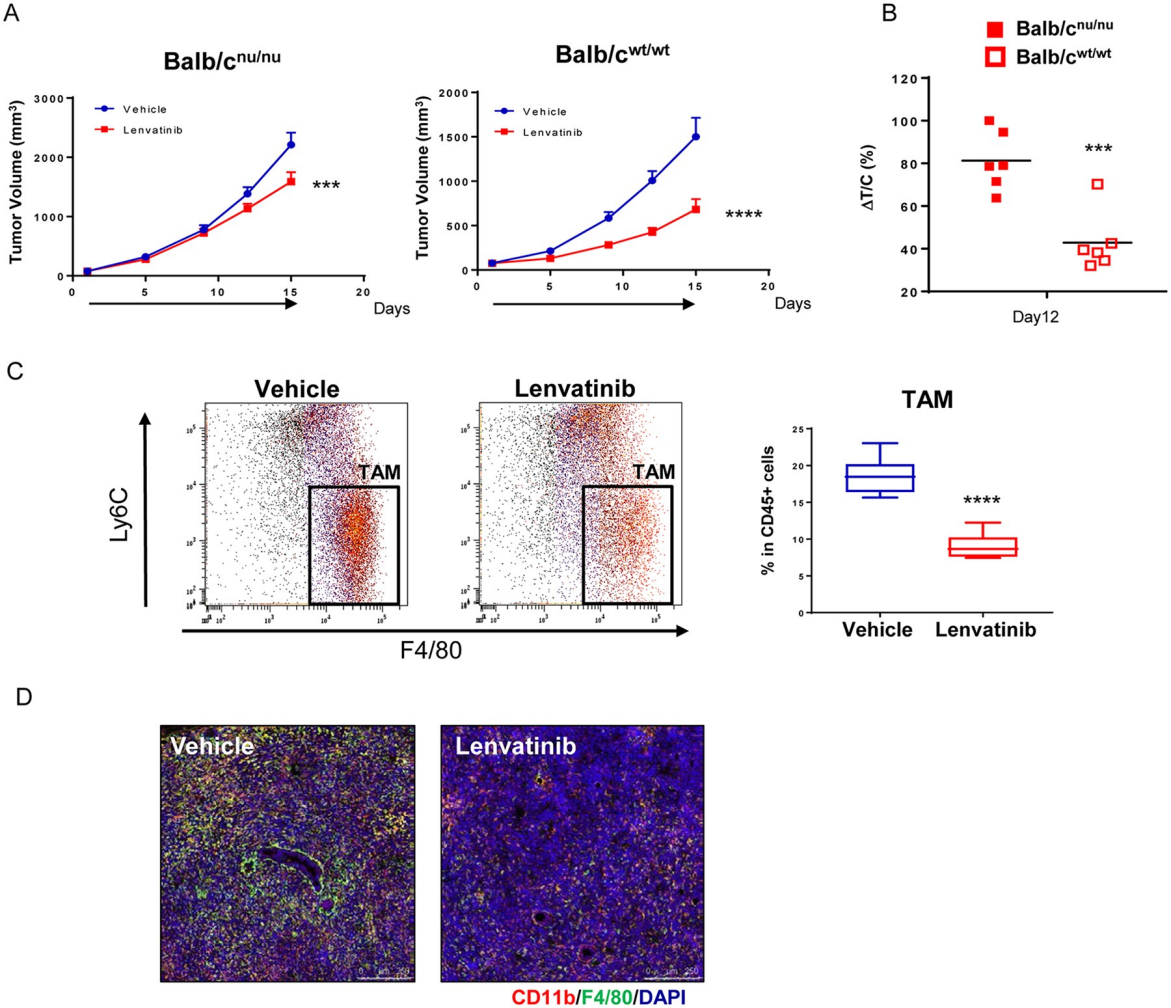
Antitumor activity of lenvatinib in immunocompetent and immunodeficient mice in the CT26 model. A. Immunodeficient mice (Balb/c^nu/nu^) and immunocompetent mice (Balb/c^wt/wt^) inoculated with the CT26 cells were randomized into groups of 6 mice with an average tumor volume size (Day 1 mean TV: Balb/c^nu/nu^ mice, 76.7 mm^3^; Balb/c^wt/wt^ mice, 80.0 mm^3^), and were then treated with vehicle (blue circles) or 10 mg/kg lenvatinib (red squares) once daily (black arrows). Error bars indicate the SEM. B. The values of ΔT/C (%) were plotted for Balb/c^nu/nu^ mice (red-filled squares) and Balb/c^wt/wt^ mice (red-open squares). ****, *P*<0.0001; ***, *P*<0.001; Dunnett’s test vs vehicle, ***P<0.001, Dunnett’s test vs. Balb/c^nu/nu^ mouse (n = 6). C. Mice, inoculated with CT26 cells, were randomized on Day 9 after CT26 inoculation with an average tumor volume size (Day 1 mean TV, 138 mm^3^) into groups of 6 or 7 mice, and were then treated with vehicle (blue bar) or 10 mg/kg lenvatinib (red bar). TILs were isolated on Day 8 after the start of treatment, and the percentage of TAMs gated by using Ly6C^−^ F4/80^+^ (black square) in the CD11b^+^ Ly6G^−^ population was analyzed and plotted. ****, *P*<0.0001, unpaired t-test vs. vehicle (*n* = 6 or 7). D. Immunohistochemical analysis of the TAM population in CT26 tumor tissues. CD11b is stained red, F4/80 is green, and DAPI is blue.

To investigate the immunomodulatory activity of lenvatinib, in addition to its known antiangiogenetic activity [8], we compared the antitumor activity of lenvatinib in immunocompetent mice (Balb/c^wt/wt^ mice) with that in immunodeficient mice (Balb/c^nu/nu^ mice) by using the CT26 mouse colon carcinoma model (CT26 model) and BNL 1ME A.7R.1 mouse HCC cells (BNL model). Lenvatinib (10 mg/kg) inhibited tumor growth in both mouse models compared with vehicle treatment, but the tumor growth of the CT26 isograft was delayed significantly in Balb/c^wt/wt^ mice compared with Balb/c^nu/nu^ mice (Fig 1A and 1B). Lenvatinib at 3 and 10 mg/kg also inhibited tumor growth of the BNL model in Balb/c^nu/nu^ mice, but it caused shrinkage of BNL tumors in Balb/c^wt/wt^ mice only (S2 Fig). These findings indicate that lenvatinib has more potent antitumor activity in the immunocompetent tumor microenvironment.

To investigate effects of lenvatinib on tumor-infiltrating lymphocytes (TILs), we performed a single-cell gene expression analysis of TILs (CD45^+^ cells) in BNL tumor tissues. We collected and sequenced RNA from 301 and 220 cells of non-treated and lenvatinib-treated tumors, respectively. tSNE analysis showed that the total TILs (521 cells) from the lenvatinib-treated and vehicle groups could be divided into three immune cell populations. Compared with non-treatment, lenvatinib increased the number of immune cells in the C1 category but decreased the number of cells in the C3 category (S3A and S3B Fig). The gene markers of immune cell populations indicated that T cell, NK cell, and cytotoxic cell markers were expressed by the C1-categorized cells. Neutrophil markers were expressed by the C2-categorized cells. Macrophage markers such as Cx3cr1, Mrc1 and Csf1r were expressed by most of the C3-categorized cells (S3C Fig). These results suggest that lenvatinib decreased the TAM population, but increased the T, NK, and cytotoxic cell populations.

Consistent with the results of the single-cell analysis, flow cytometric analysis indicated that the TAM population (gated as CD45^+^ CD11b^+^ Ly6G^−^ Ly6C^−^ F4/80^+^) was significantly decreased by lenvatinib treatment compared with vehicle treatment in both the CT26 model (Fig 1C) and the BNL model (S4A Fig). In addition, immunohistochemical analysis showed that lenvatinib treatment reduced the number of CD11b^+^ F4/80^+^ double-positive cells in the tumor (indicated in yellow in Fig 1D and S4B Fig). These results indicate that lenvatinib decreases the TAM population in both the CT26 and BNL models.

In the CT26 model, the effect of TAM depletion on T cell activation was examined by using an anti-CSF1R antibody. In the presence of the anti-CSF1R antibody, Prf1 and GzmB expression increased, whereas the expression of TAM-related genes, such as Csf1r, Cx3cr1 and Itgam, decreased (S5 Fig). These data suggest that reduced TAM infiltration by lenvatinib might cause activation of CD8^+^ T cells.

### Attenuation of the antitumor activity of lenvatinib upon loss of CD8^+^ T cell activation in the CT26 model

**Fig 2:**
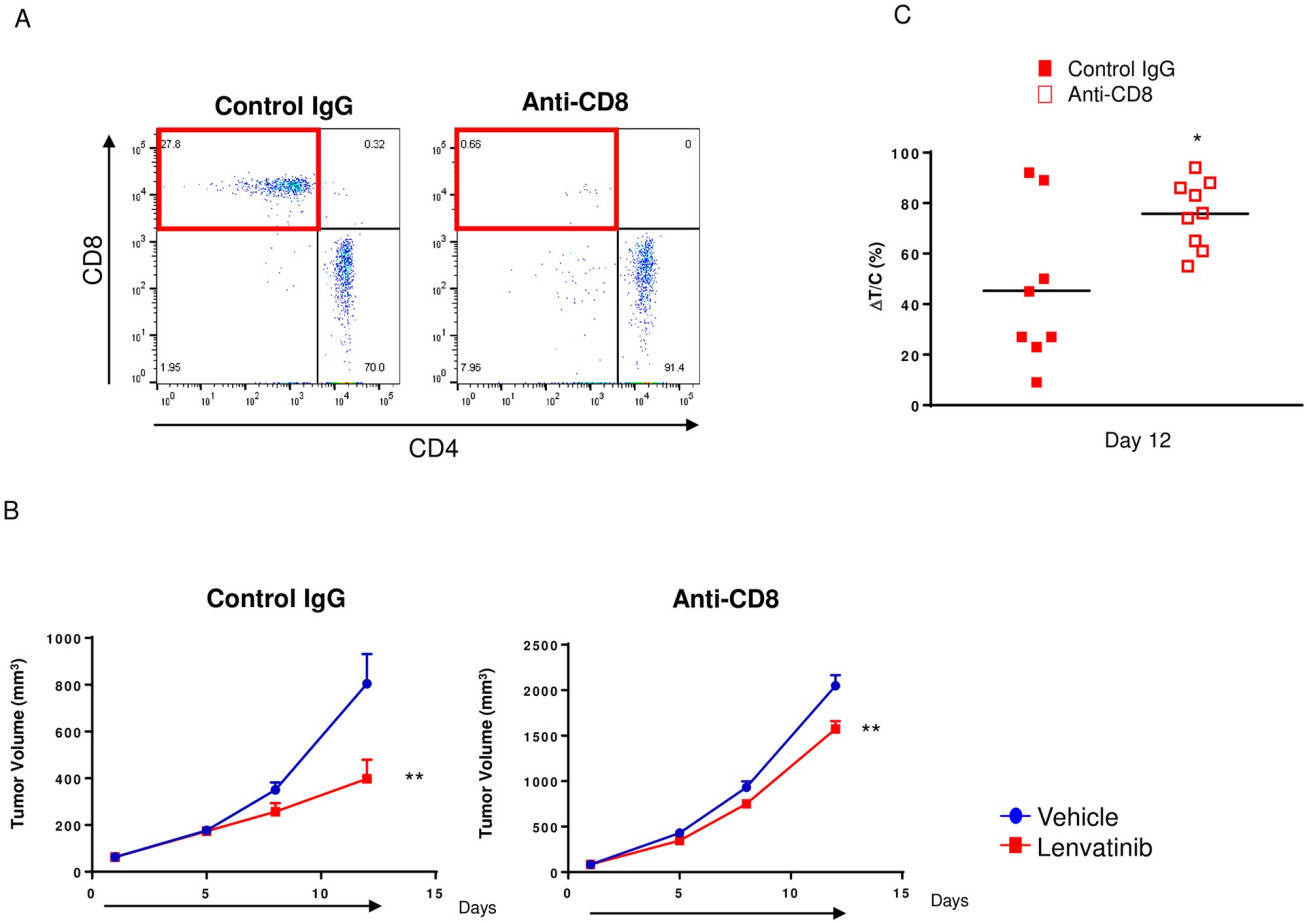
Antitumor activity of lenvatinib under CD8^+^ T cell-depleted conditions in the CT26 model. Mice, inoculated with CT26 cells, were treated with anti-CD8 antibody or control IgG on Day 6 and twice weekly thereafter. A. For flow cytometric analysis, the percentage of CD8^+^ T cells in the blood was determined. B. Two days after the first injection of anti-CD8 antibody or control IgG, mice were randomized into groups of 8 or 9 with an average tumor volume size (Day 1 mean TV: anti-CD8 antibody, 65.9 mm^3^; control IgG, 49.3 mm^3^), and then treated with vehicle (blue circles) or 10 mg/kg lenvatinib (red squares), indicated by the black arrow. Error bars represent the SEM. C. The percentage of ΔT/C (tumor size of each mouse treated with lenvatinib divided by the average tumor size of vehicle-treated mice) is shown for the control IgG (red-filled squares) and anti-CD8 antibody (red-open squares) treatment groups. **, *P*<0.01, Dunnett’s test vs. vehicle or control IgG (*n* = 8 or 9).

To evaluate whether the antitumor activity of lenvatinib was dependent on CD8^+^ T cell activation, we compared the antitumor activity of lenvatinib with and without CD8^+^ T cells in the CT26 model by using an anti-CD8 antibody in Balb/c^wt/wt^ mice. We used flow cytometry to confirm that CD8^+^ T cell percentage were decreased as a result of antibody depletion (Fig 2A). Compared with vehicle treatment, lenvatinib significantly inhibited tumor growth despite CD8^+^ T cell depletion; the antitumor activity of lenvatinib was reduced under these CD8^+^ T cell-depleted conditions. The ΔT/C values of lenvatinib in control IgG-treated mice were significantly smaller than those in CD8^+^ T cell-depleted Balb/c^wt/wt^ mice (Fig 2B and 2C).

### Antitumor activity of lenvatinib plus anti-PD-1 in the CT26 model

**Fig 3:**
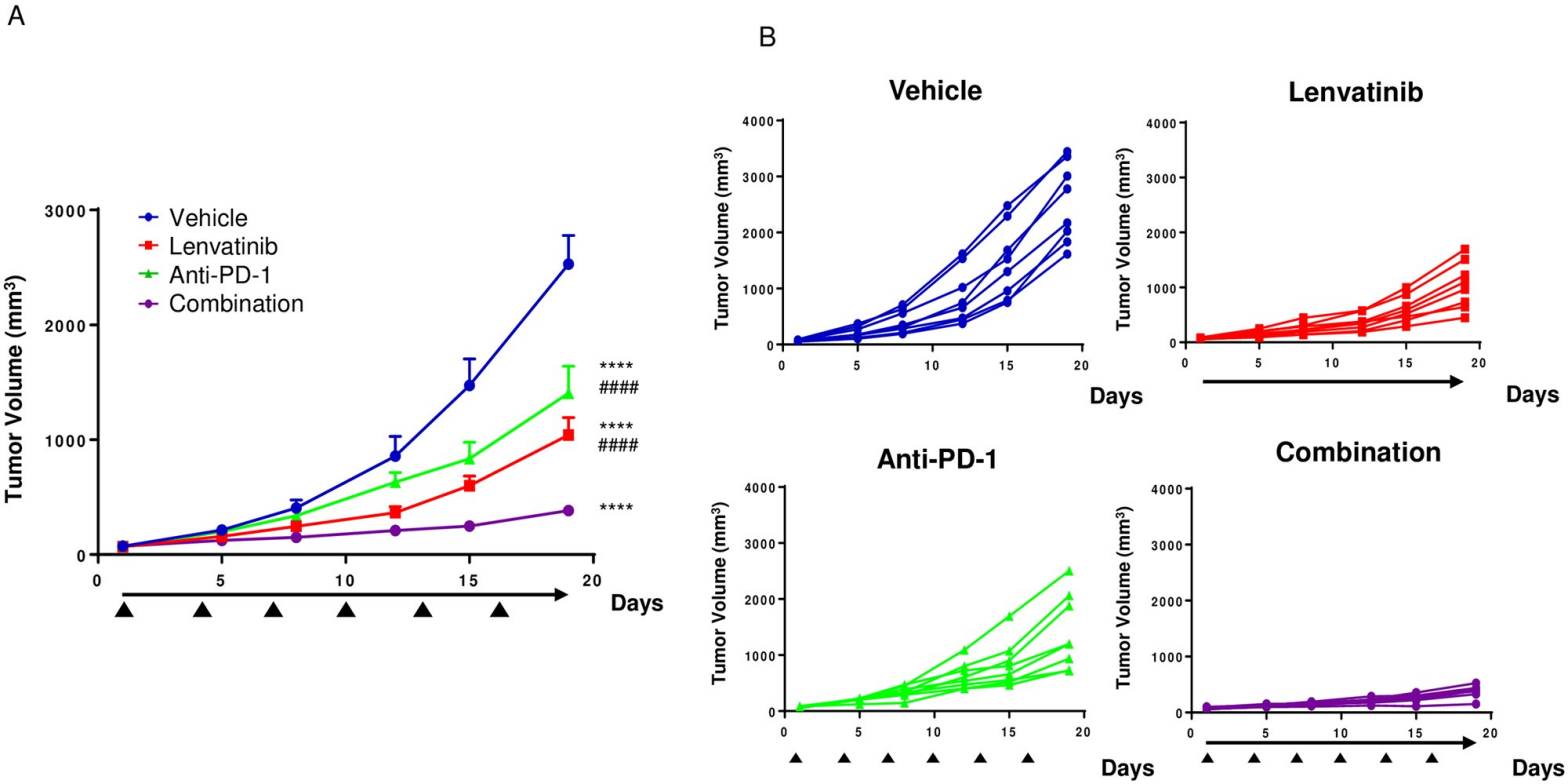
Antitumor effects of lenvatinib treatment alone or in combination with anti-PD-1 in the CT26 model. A. Mice were inoculated with CT26 cells and randomized into groups of 8 with an average tumor volume size (Day 1 mean TV, 32.6 mm^3^) and were then treated with vehicle (blue circles), 10 mg/kg lenvatinib (red squares) once daily, anti-PD-1 at 200 μg/mouse (green triangles) once every 3 days, or a combination of lenvatinib plus anti-PD-1 (purple circles). Lenvatinib treatment is indicated by the black arrow; anti-PD-1 treatment is indicated by black triangles. B. Tumor volumes for individual mice are shown for each treatment group. Error bars represent the SEM. **** *P*<0.0001, Dunnett’s test vs. vehicle on Day 19; ## *P*<0.01, #### *P*<0.0001 vs. combination.

We investigated the combined antitumor activity of lenvatinib plus anti-PD-1 in the CT26 model. Treatment with lenvatinib or anti-PD-1 alone significantly inhibited the in vivo tumor growth of CT26 isografts compared with vehicle treatment (Fig 3A and 3B). Combined lenvatinib plus anti-PD-1 treatment significantly suppressed tumor growth compared with either treatment alone. Similarly, lenvatinib or anti-PD-1 alone significantly delayed tumor growth compared with vehicle treatment in the B16-F10 mouse melanoma syngeneic model, and lenvatinib plus anti-PD-1 combination therapy was more potent than either treatment alone (S6 Fig).

### Effect of lenvatinib plus anti-PD-1 on the tumor microenvironment in the CT26 model

**Fig 4:**
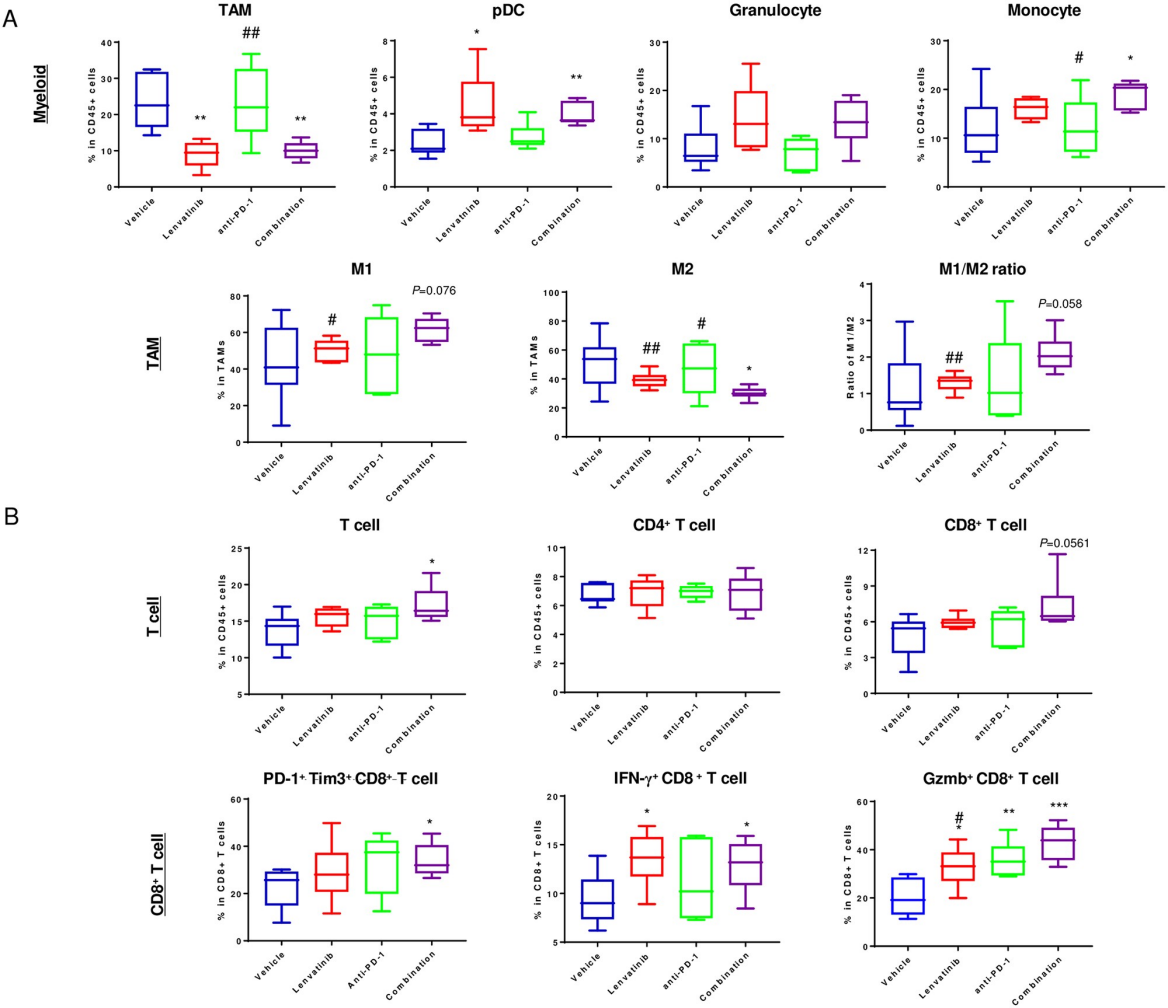
Immune cell population analysis of lymphocytes in the CT26 tumor tissues. A, Percentages of myeloid and TAM cell populations. Upper panel: TAMs, pDCs, granulocytes, and monocytes. Lower panel: M1, M2 in CD45^+^ cells, and the M1/M2 ratio. B. Upper panel: T cells, CD4^+^ T cells, and CD8^+^ T cells. Lower panel: PD-1^+^ Tim3^+^ CD8^+^ T cells, IFN-γ^+^ CD8^+^ T cells, and GzmB^+^CD8^+^ T cells. The gating strategy is shown in S1 Fig. *P <0.05, **P <0.01, ***P<0.001, unpaired t-test vs. vehicle; #*P*<0.05, unpaired t-test vs. combination (n = 6).

Next, we investigated the effect of lenvatinib plus anti-PD-1 on the tumor microenvironment, including tumor blood vessels and immune cell populations by using immunohistochemical analysis and flow cytometric analysis, respectively, on Day 8 after each treatment. First, we evaluated effects on microvessel density (areas rich in CD31-positive blood vessels) in CT26 isografts. Lenvatinib treatment alone and the combination treatment decreased the number of tumor blood vessels, but anti-PD-1 treatment alone did not (S7 Fig). The antiangiogenesis activity of lenvatinib was comparable to that of the combination treatment in the CT26 model, suggesting an alternative mechanism for the antitumor activity of lenvatinib plus anti-PD-1. Using flow cytometry, we compared changes in the percentages of immune cell populations among TILs in response to each treatment. Immune cell populations were determined as gated in S1 Fig. The TAM population was significantly decreased by combination treatment and lenvatinib treatment alone at equivalent levels. Among the TAMs, lenvatinib plus anti-PD-1 decreased the M2 population (I-A/I-E^low^), and the ratio of M1 (I-A/I-E^high^)/M2 tended to be increased by lenvatinib plus anti-PD-1 combination treatment. In other myeloid cells, lenvatinib increased the percentage of plasmacytoid dendritic cells (pDCs), and combination treatment increased the pDC and monocyte populations (Fig 4A). Combination treatment also increased the percentage of T cells, especially CD8^+^ T cells. PD-1^+^ Tim3^+^ CD8^+^ T cells were also increased in the combination treatment group compared with vehicle treatment. IFN-γ^+^ CD8^+^ T cells were increased in the lenvatinib and combination treatment groups compared with vehicle treatment. GzmB^+^ CD8^+^ T cells were increased in each single-treatment group compared with vehicle treatment, and combination treatment further increased the number of GzmB^+^ CD8^+^ T cells compared with lenvatinib treatment alone. These results suggest that CD8^+^ T cells and their cytotoxic activity are upregulated by lenvatinib plus anti-PD-1 combination treatment in the CT26 model (Fig 4B).

### Gene co-expression network analysis to identify immune-related gene sets modulated in response to the combined treatment of lenvatinib plus anti-PD-1

**Fig 5:**
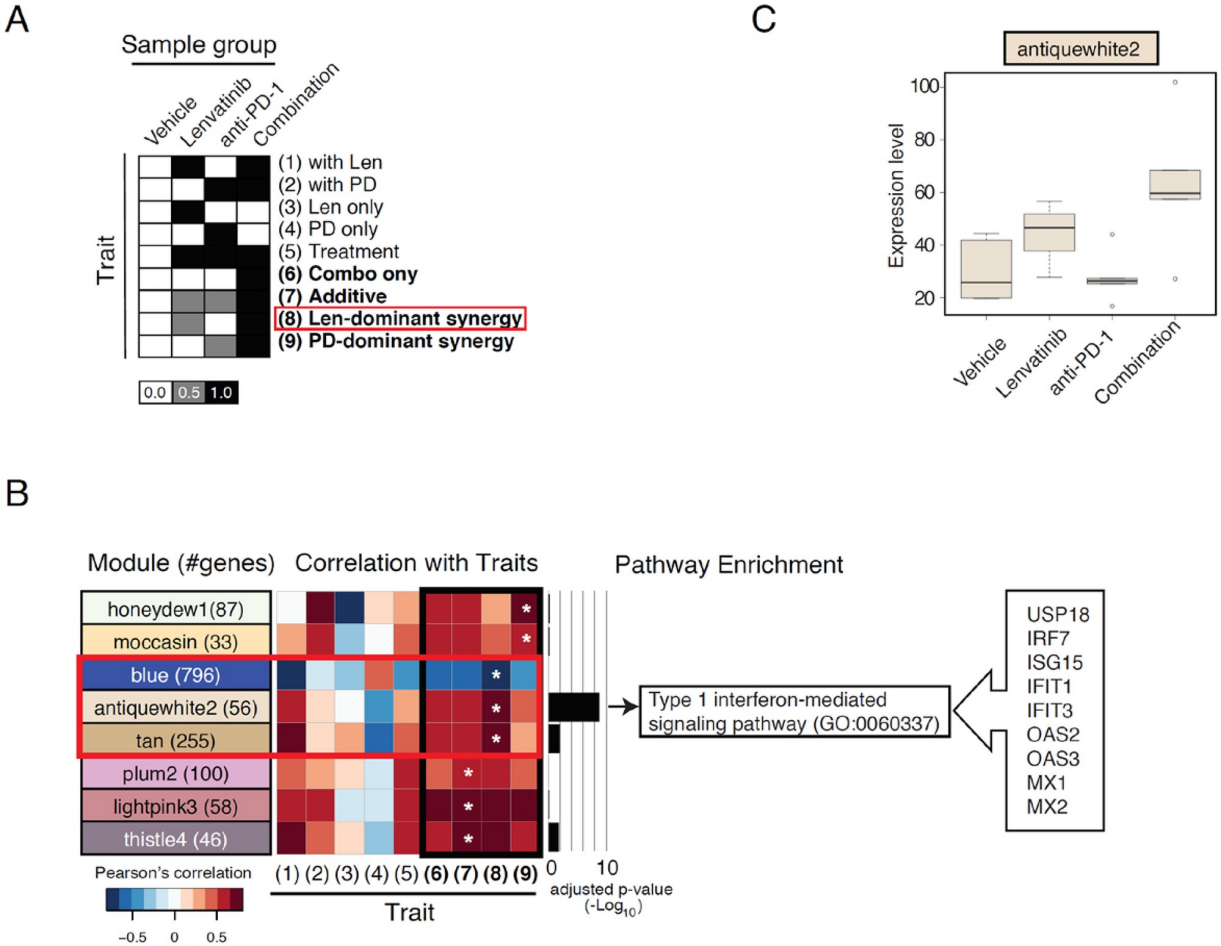
Weighted gene co-expression network analysis (WGCNA) to identify gene subsets modulated by combination treatment. RNA-Seq analysis of tumor tissues followed by WGCNA was performed by using CT26 tumor tissues. A. Defined sample traits, each of which reflects a pattern of expression as indicated in the text. Gene subsets that correlate with one of traits (6) through (9) are considered to be possible target gene sets of the ‘potentiating effect’ of the two drugs. B. Modules from the WGCNA selected for trait correlation analysis. The modules highly correlated with one of the modules in A (r>0.65 and *P*<0.01) are shown with the number of genes included in each module in parentheses. The correlation strength of each module with each trait is shown in the heatmap where the most highly correlated trait is indicated by an asterisk. The significance (FDR-adjusted *P* value) of the top over-represented pathway in each module is shown in the bar graph, and the significantly enriched pathway is indicated (FDR-adjusted *P* value <0.05). The genes in the antiquewhite2 module represented in the type-I IFN signaling pathway are shown in the arrow box to the right. C. Eigengene expression pattern for the antiquewhite2 module.

RNA-Seq analysis followed by WGCNA and the trait correlation analysis was performed. The RNA-Seq analysis was performed by using CT26 tumor tissues from mice treated with vehicle, lenvatinib, anti-PD-1, or the combination for 1 week. The gene expression data (TPM > 1.0) of 13,847 unique genes for 20 samples comprising four sample groups (vehicle, lenvatinib [LEN], anti-PD-1 [PD], and the combination [Combo]) were divided into 170 modules labeled with distinct colors in WGCNA. Defined sample traits, each of which reflects a pattern of expression, were as follows: changes occurred in the presence of at least one of the drugs (Treatment), only in single drug treatment (Len only or PD only), in the combination and one of the two single treatments (with Len or with PD), only in the combination (Combo-only), in the combination with additive effects (Additive), and in the combination with synergistic effects (Len-dominant synergy or PD-dominant synergy) (Fig 5A). Gene subsets that correlated with one of traits (6) through (9) were considered possible targets of the ‘potentiating effect’ of the two drugs. Eight modules containing potential targets of potentiating effects were determined by WGCNA and subsequent trait analysis. These modules were selected based on the criteria that the most correlated trait was Combo-only, Additive, Len-dominant synergy, or PD-dominant synergy; the Pearson’s correlation was |r|>0.6; and the *P* value was <0.01. The significance (FDR-adjusted *P* value) of the top over-represented pathway in each module is shown in the bar graph, and the significantly enriched pathway is indicated (FDR-adjusted *P* value <0.05).

To predict the target ‘biological units’ of the potentiating modules, a pathway enrichment analysis was conducted on the module genes (Fig 5B). Interestingly, type-I IFN signaling pathway-related genes were remarkably enriched (FDR-adjusted *P* value was as low as 1.50e–9) in the antiquewhite2 module, where genes were upregulated upon lenvatinib treatment and even more so upon combination treatment (Fig 5B and 5C, S2 Table). This module includes genes for the key positive and negative regulators in the type-I interferon pathway, and these genes were also enriched in the virus response pathway and the IFN-γ-mediated signaling pathway, where component genes are overlapped in some places (S2 Table).

### Role of activation of IFN signaling in the combined antitumor activity of lenvatinib plus anti-PD-1 in the CT26 model

Based on our WGCNA, we hypothesized that activation of type-I IFN signaling by lenvatinib might induce activation of CD8^+^ T cells. Therefore, we examined the antitumor activity of anti-PD-1 with prior lenvatinib treatment. Tumor growth inhibition by anti-PD-1 with prior lenvatinib treatment for 1 week was greater than that achieved by anti-PD-1 alone, nontreatment, or nontreatment with prior lenvatinib treatment (S8 Fig). To examine if the response of tumor to lenvatinib was an important component in the response of the tumors to IFN signal activation [23], we divided the mice into two groups according to a mean of tumor sizes after 1 week of lenvatinib treatment: those with relatively lenvatinib-sensitive smaller tumors (LEN-S) and those with relatively larger tumors that were moderately sensitive to lenvatinib (LEN-L). Mice in the LEN-S group that were subsequently treated with anti-PD-1 showed greater tumor reduction than the other treatment groups (S9 Fig). To investigate signaling pathways selectively regulated in relation to tumor size after 1 week of lenvatinib treatment, we examined upstream regulators using IPA based on RNA-Seq data from LEN-S and LEN-L tumors harvested on Day 8. The analysis showed that VEGFA was one of the top-ranked molecules among the inhibitory upstream regulators in both groups. In contrast, type-I and type-II IFN-related molecules were top-ranked activate upstream regulators only in LEN-S. These data suggest that IFN pathways play important roles in lenvatinib treatment-sensitive tumors (S3 Table). Heatmap data showing T cell related genes [24] or type-I IFN-related gene expression in LEN-S and LEN-L also indicated that T cell activation and type-I IFN-related gene expression were higher in LEN-S compared with LEN-L (S10 Fig).

**Fig 6:**
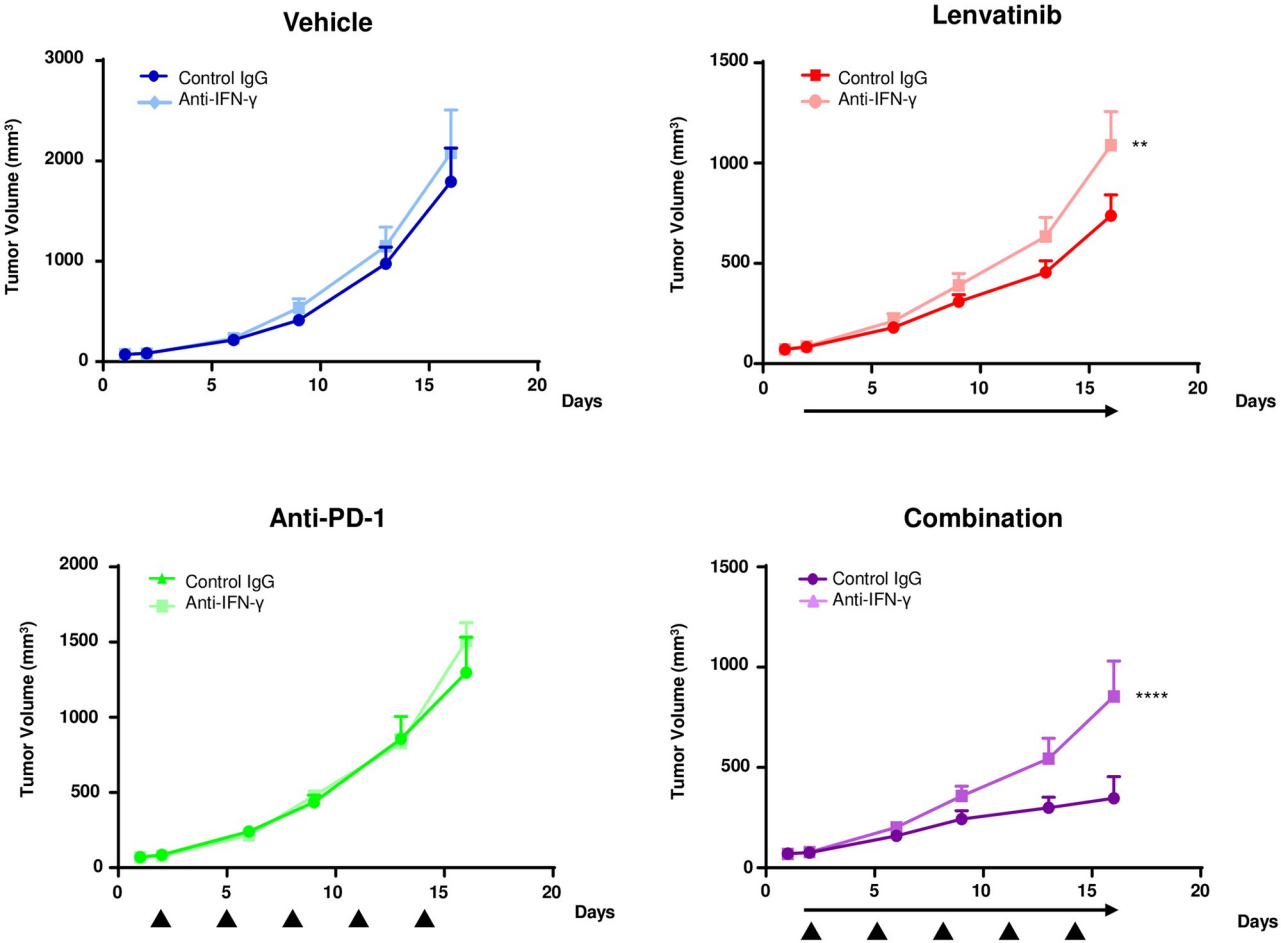
Antitumor activity of lenvatinib under IFN-γ-inhibited conditions in the CT26 model. A. Mice, inoculated with CT26 cells, were treated with anti-IFN-γ antibody or control IgG on Day 8 after CT26 inoculation and twice weekly thereafter. The mice were randomized into groups of 7 with an average tumor volume size (Day 1 mean TV 71.1 mm^3^). The day after the first injection of anti-IFN-γ antibody or control IgG, mice were treated with vehicle (control IgG, dark blue; anti-IFN-γ antibody, light blue), 10 mg/kg lenvatinib (control IgG, dark red; anti-IFN-γ antibody, light red), anti-PD-1 (control IgG, dark green; anti-IFN-γ antibody, light green) or combination (control IgG, dark purple; anti-IFN-γ antibody, light purple). Lenvatinib treatment is indicated by the black arrow, and anti-PD-1 treatment is indicated as black triangles. Error bars represent the SEM. **P<0.01, ****P<0.0001, Dunnett’s test vs. control IgG (n = 7).

Finally, we examined the roles of IFN-γ in the antitumor activity of lenvatinib plus anti-PD-1 in the CT26 model by using an anti-IFN-γ antibody. The antitumor activity of vehicle and anti-PD-1 alone was unaffected by IFN-γ inhibition, but that of lenvatinib alone and in combination with anti-PD-1 was significantly inhibited by IFN-γ inhibition. These data demonstrate that the antitumor activity of lenvatinib plus anti-PD-1 combination treatment is dependent on lenvatinib’s dominant activation of the IFN-γ signaling pathway (Fig 6).

## Discussion

Recently, we reported that the immunomodulatory activity of lenvatinib leads to potent antitumor activity in the Hepa1-6 mouse HCC model [25]. In the current study, instead of the highly T cell-inflamed Hepa1-6 tumor model, we used the CT26 tumor model, a frequently used syngeneic mouse model demonstrating moderate T cell-inflamed tumor, and the BNL model, a syngeneic non-T cell inflamed HCC model. Consistent with our results using the Hepa1-6 model, single-cell analysis of TILs in BNL tumors showed that lenvatinib reduced immune cell populations, including those with macrophage markers. Flow cytometric analyses showed decreased TAM populations in both the CT26 and BNL model (Fig 1 and S3 Fig). These findings suggest that lenvatinib has immunomodulatory activity against both T cell-inflamed and non T cell-inflamed tumor microenvironments. In contrast, anti-PD-1 did not affect the myeloid populations. These data suggest that the antitumor activity of the combination treatment a lenvatinib plus anti-PD-1 would be dependent on the activity of lenvatinib against the myeloid cell populations.

The antitumor immune response generally varies depending on the duration of treatment, but recent findings suggest that analysis approximately 1 week is sufficient to reveal changes in immune cell populations [26, 27]. In our current study, flow cytometric analysis on Day 8 also showed that lenvatinib plus anti-PD-1 combination treatment increased T cell numbers, especially those of CD8^+^ T cells and GzmB^+^-activated CD8^+^ T cells. These results are supported by our finding that TAM depletion by the use of an anti-CSF1R antibody induced activation of CD8^+^ T cells, as indicated by upregulation of Prf1 and GzmB (S5C Fig). Our results thus reveal that lenvatinib is not only a potent angiogenesis inhibitor but also an effective immunomodulator whose functions differ from those of immune checkpoint inhibitors.

Voron *et al*. reported that VEGFA produced from cancer cells and stromal cells upregulated the expression level of PD-1 and other inhibitory checkpoints involved in CD8^+^ T cell exhaustion, and that VEGFR inhibitors inhibit immune regulatory function by targeting VEGFR signaling [28]. The gene expression patterns of angiogenesis factors such as VEGFA are major gene expression signatures in clinical samples from anti-PD-1-nonresponsive patients [29].

The VEGFR signaling pathway also plays important roles in the migration of monocytes from bone marrow into tumors [30–32]. In addition, the FGF2-mediated FGFR1 signaling pathway regulates the survival and migration of TAMs in the tumor microenvironment in esophageal cancer [33]. Therefore, these reports suggested that lenvatinib may increase antitumor immunity through decrease of the TAM population by inhibiting the VEGFR and FGFR signaling pathways.

In the current study, WGCNA using the RNA-Seq data revealed that the IFN signaling pathway was extracted as Len-dominant synergistic pathway. In addition, molecules involved in the type-I and type-II IFN signaling pathways were the main lenvatinib-activated upstream regulators in mice with small tumors (S3 Table). The type-II IFN, IFN-γ, is a key cytokine in the stimulation of a host antitumor immune response, including antigen presentation and T cell trafficking [34]. Type-I IFN signal activation promotes the death of cancer cells and restricts tumor growth by altering the immune microenvironment [23, 35, 36]. Moreover, the efficacy of PD-1 blockade is dependent on the activation status of the IFN pathway [23]. Therefore, the activation of type-I and -II IFN signaling in tumor after lenvatinib treatment could be preferable for anti-PD-1 antibody therapy.

In our previous report, we showed that the dual inhibitory activity of lenvatinib against both VEGF and FGF induced broad-spectrum antitumor activity due to the antiangiogenesis effects [8]. These antiangiogenetic effects convert the immunosuppressive status of the tumor microenvironment to a pro-tumor milieu and lead to priming of increased IFN-γ production by cytotoxic T cells [37]. Together, these reports suggest that lenvatinib’s strong antitumor activity associated with angiogenesis inhibition is related to the activate IFN signaling in the tumor microenvironment.

In contrast, rather than IFN signaling-related molecules, CTLA-4 emerged as an upstream regulator in tumors that were moderately sensitive to lenvatinib (LEN-L). In a Phase I study, combined treatment with anti-VEGFA antibody and anti-CTLA-4 antibody achieved favorable outcomes in patients with metastatic melanoma [38]. For tumors moderately sensitive to lenvatinib, the addition of anti-CTLA-4 antibody may yield an effective combination therapy.

Our results suggest that two inhibitory mechanisms––antiangiogeneis and anti-TAM activity––are responsible for the antitumor and immunomodulatory activities of lenvatinib. Currently, Phase 1b/2 trials of lenvatinib plus pembrolizumab in patients with selected solid cancers (NCT02501096/NCT03006926), a Phase 3 trial of lenvatinib in combination with pembrolizumab in patients with advanced renal cell cancer (NCT02811861) and Phase 1b of lenvatinib plus nivolumab in patients with HCC (NCT03418922) are underway. Further analyses are needed to precisely delineate the contributions of lenvatinib-induced VEGFR and FGFR inhibition to the suppression of TAM infiltration and IFN signaling activation as a mechanism of action in this combination therapy.

## Conclusion

Our findings in this study revealed that lenvatinib, a potent VEGFR and FGFR signaling inhibitor, shows more potent antitumor activity when combined with PD-1 blockade by decreasing TAM numbers, thereby affecting antitumor immune responses. In addition, this combination treatment enhanced the activation of the IFN signaling pathway. These findings demonstrate the mechanism of action of lenvatinib alone and in combination with PD-1 blockade. Our results provide a scientific rationale for evaluating lenvatinib plus PD-1 blockade as combination therapy in clinical trials to improve cancer immunotherapy.

## Supporting information

S1 Fig Gating strategy for the flow cytometric analysis in myeloid and T cell panels. Immune cell populations analyzed in the Myeloid panel (TAMs, monocytes, granulocytes, pDCs, M1, and M2) and T cell panel (T cells, CD4^+^ T cells, CD8^+^ T cells, PD-1^+^ Tim3^+^ CD8^+^ T cells, IFN-γ^+^ CD8^+^ T cells, GzmB^+^ CD8^+^ T cells) shown in Figs 1, 2 and 4 were gated as indicated in the sequence of blue arrows.
(TIF)

S2 Fig Antitumor activity of lenvatinib in immunocompetent and immunodeficient mice in the BNL model. Immunodeficient mice (Balb/c^nu/nu^) and immunocompetent mice (Balb/c^wt/wt^) inoculated with BNL cells were randomized into groups of 5 with an average tumor volume size of 155 mm^3^ (Balb/c^wt/wt^ mouse) or 170 mm^3^ (Balb/c^nu/nu^ mouse) (Day 1) and then treated with vehicle (blue circle), 3 mg/kg lenvatinib (orange square), or 10 mg/kg lenvatinib (red square) once daily, indicated by the black arrow. Error bars represent the SEM. ****P<0.0001, Dunnett’s test vs. vehicle (n = 5).
(TIF)

S3 Fig Single cell and RNA-Seq analyses of tumor-infiltrating lymphocytes in the BNL model. A. Single cells (521) are depicted in the tSNE two-dimensional space, where each plot represents each of the cells. Vehicle-treated cells are shown in cyan on the left; lenvatinib-treated cells are shown in red on the right. Cells were separated into three distinct clusters (C1: blue, C2: green, and C3: pink). B. Comparison of the changes in the proportions of cells in the three clusters between vehicle- and lenvatinib-treated cells. C. Expression levels of eight cell-specific marker genes.
(TIF)

S4 Fig Immune cell population analysis in the BNL model. A and B. Representative profiles from flow cytometric analysis showing TAMs gated as a CD45^+^CD11b^+^Ly6G^-^Ly6C^-^F4/80^+^ population in tumors derived from vehicle- or 10 mg/kg lenvatinib-treated mice are shown. The percentage of the TAM population among the CD45^+^ cells is plotted. **P<0.01, unpaired t-test (n = 10). C. Representative images from immunohistochemical analyses showing CD11b (red) and F4/80 (green). TAMs are stained yellow.
(TIF)

S5 Fig Immunomodulatory activity of anti-CSF1R antibody in the CT26 model. A. Tumor tissues were harvested on Day 8, and TAM depletion by the anti-CSF1R antibody was confirmed by flow cytometry. B. Gene expression profiles of Csf1r, Cx3cr1, Itgam, Tgfb1, Il15, Il6, Prf1, GzmB, and Ifng in tumor tissues from control IgG-treated mice (blue) and anti-CSF1R antibody-treated mice (orange) in quantitative PCR analysis. *P<0.05, ***P<0.001, unpaired t-test (n = 6).
(TIF)

S6 Fig Antitumor activity of lenvatinib alone or in combination with anti-PD-1 in the B16-F10 model. A. Mice were inoculated with B16-F10 cells and randomized into groups of 10 with an average tumor volume size of 71 mm^3^ (Day 1) and then treated with vehicle (blue circle), 10 mg/kg lenvatinib (red square) once daily, anti-PD-1 at 500 μg/mouse (green triangle) once every 3 days, or the combination (purple triangle). Lenvatinib treatment is indicated by the black arrow, and anti-PD-1 treatment is indicated as black triangles. B. Changes in tumor size for individual mice are shown for each group. *P<0.05, ****P<0.0001, Dunnett’s test vs. vehicle; ##*P*<0.01, ####*P*<0.0001 vs. combination.
(TIF)

S7 Fig Immune cell population analysis in the CT26 model. A. Representative images from immunohistochemical analysis showing CD31 (red) and DAPI (blue). B. The number of CD31-stained cells and the area of the tumor tissue slice were determined for each mouse by using HALO; the CD31^+^ cell number was then divided by the tumor tissue area (mm^2^) and plotted. *P<0.05, ***P<0.001, Dunnet’s test vs. vehicle.
(TIF)

S8 Fig Antitumor activity of anti-PD-1 with prior lenvatinib treatment. A. Mice were inoculated with CT26 cells and randomized into 3 groups with an average tumor volume of 78.4 mm^3^ (Day 1) as follows: nontreatment, 8 mice; anti-PD-1 treatment, 8 mice; and lenvatinib treatment, 32 mice. Mice were then treated with nontreatment (blue circle) or anti-PD-1 at 200 μg/mouse (green triangle) once every 3 days. The lenvatinib-treated mice were further randomized into 2 groups of 16 mice on Day 8 at an average tumor volume of 212 mm^3^ and then treated with anti-PD-1 at 200 μg/mouse (pink circle) once every 3 days or nontreatment (orange square). Lenvatinib treatment is indicated by the pink arrow and anti-PD-1 treatment is indicated by the green arrow. Nontreatment is indicated by the blue-framed arrow. B. Changes in tumor size for individual mice are shown for each treatment group. Error bars represent the SEM. *P<0.05, ****P<0.0001, Dunnett’s test vs. vehicle; ###*P*<0.001, ####*P*<0.0001, vs. combination.
(TIF)

S9 Fig Antitumor activity of anti-PD-1 based on tumor volumes with prior treatment of lenvatinib for 1 week. Using the same data as those used in the legend to S8 Fig, lenvatinib-treated mice were further divided into two groups based on the tumor volume on Day 8: the lenvatinib-sensitive relatively small tumor group (LEN-S; mean TV, 127 mm^3^; n = 16) and the lenvatinib moderate sensitive relatively large tumor group (LEN-L; mean TV, 296 mm^3^; n = 16). Tumor growth was then re-analyzed according to anti-PD-1 treatment (n = 8) or nontreatment (n = 8) for both the LEN-S and LEN-L group. Tumor growth in mice treated with nontreatment (blue circle), anti-PD-1 (green), LEN-S followed by anti-PD-1 (red) or nontreatment (dark pink), and LEN-L followed by anti-PD-1 (orange) or nontreatment (pink). B. Changes in tumor volumes for individual mice are shown for each treatment group. Error bars represent the SEM. ****P<0.0001, ****P<0.0001, Dunnett’s test vs. LEN-L followed by anti-PD-1; ##*P*<0.01, ###*P*<0.001, ####*P*<0.0001, vs. LEN-S followed by anti-PD-1.
(TIF)

S10 Fig Gene expression profiles of tumor tissues of the LEN-S and LEN-L groups in the CT26 model. Using RNA-Seq data from the LEN-S and LEN-L tumors, gene expression levels of T cell-related genes and type-I IFN-related genes compared with vehicle were plotted as heatmaps. The gene expression level indicator is shown at the bottom right.
(TIF)

S1 Table List of antibodies for flow cytometry analysis. (TIF)

S2 Table The properties of the antiquewhite2 module in S3 Fig. The module genes and their correlation with the module eigengene and enriched biological pathways.
(TIF)

S3 Table Upstream regulators of gene expression changes in LEN-S and LEN-L tumors compared with nontreated tumors in S9 Fig. The top ten activated or inhibited upstream regulators in LEN-S and LEN-L compared with vehicle are listed.
(TIF)
