# Supplementary figures and images for: Lenvatinib plus anti-PD-1 antibody combination treatment activates CD8+ T cells through reduction of tumor-associated macrophage and activation of the interferon pathway
Source: PLoS One. 2019 Feb 27;14(2):e0212513. doi: 10.1371/journal.pone.0212513 (PMC6392299; doi:10.1371/journal.pone.0212513)

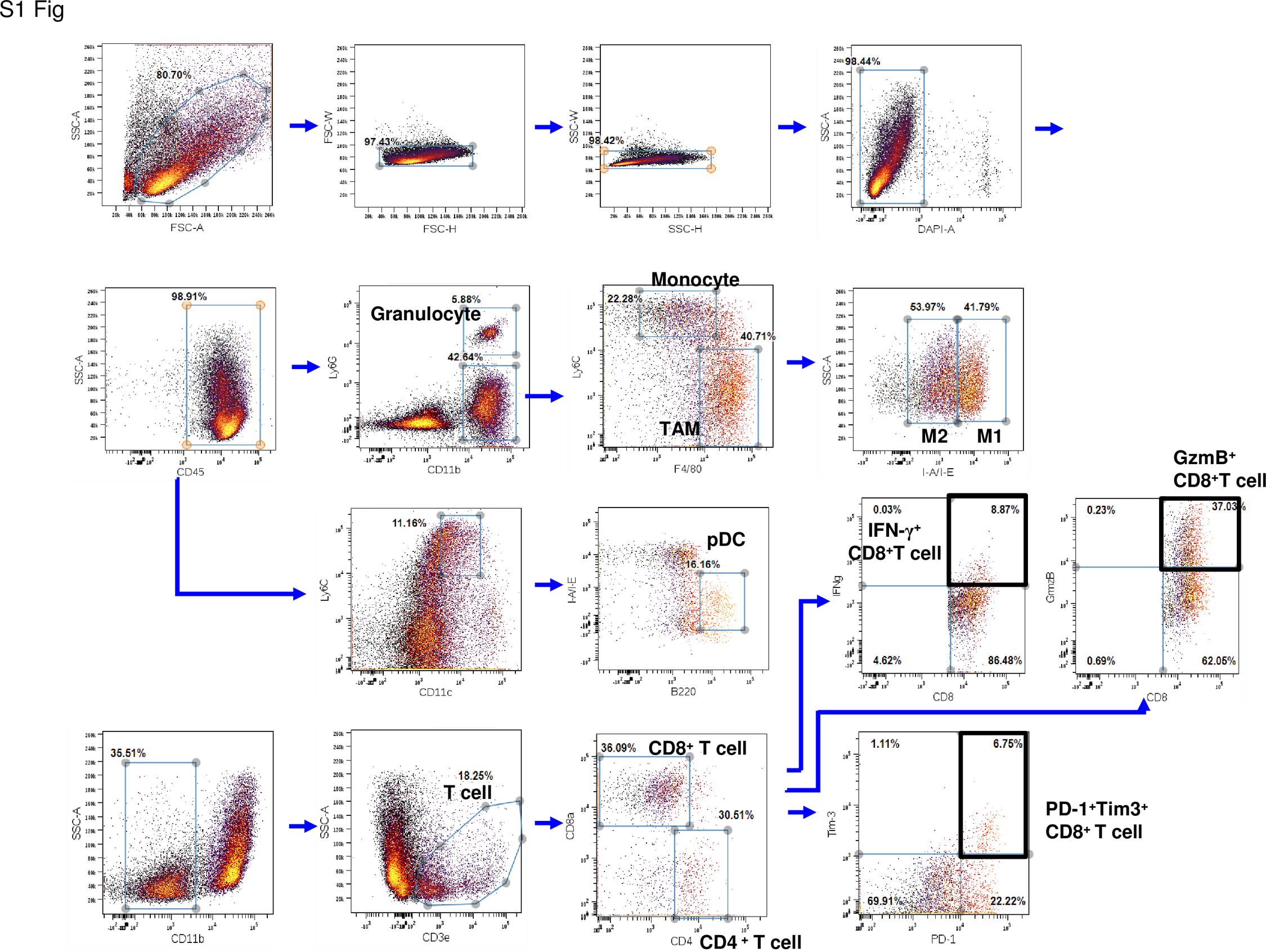

Supplement: S1 Fig — Immune cell populations analyzed in the Myeloid panel (TAMs, monocytes, granulocytes, pDCs, M1, and M2) and T cell panel (T cells, CD4+ T cells, CD8+ T cells, PD-1+ Tim3+ CD8+ T cells, IFN-γ+ CD8+ T cells, GzmB+ CD8+ T cells) shown in Figs 1, 2 and 4 were gated as indicated in the sequence of blue arrows. (TIF) [file pone.0212513.s001.tif]

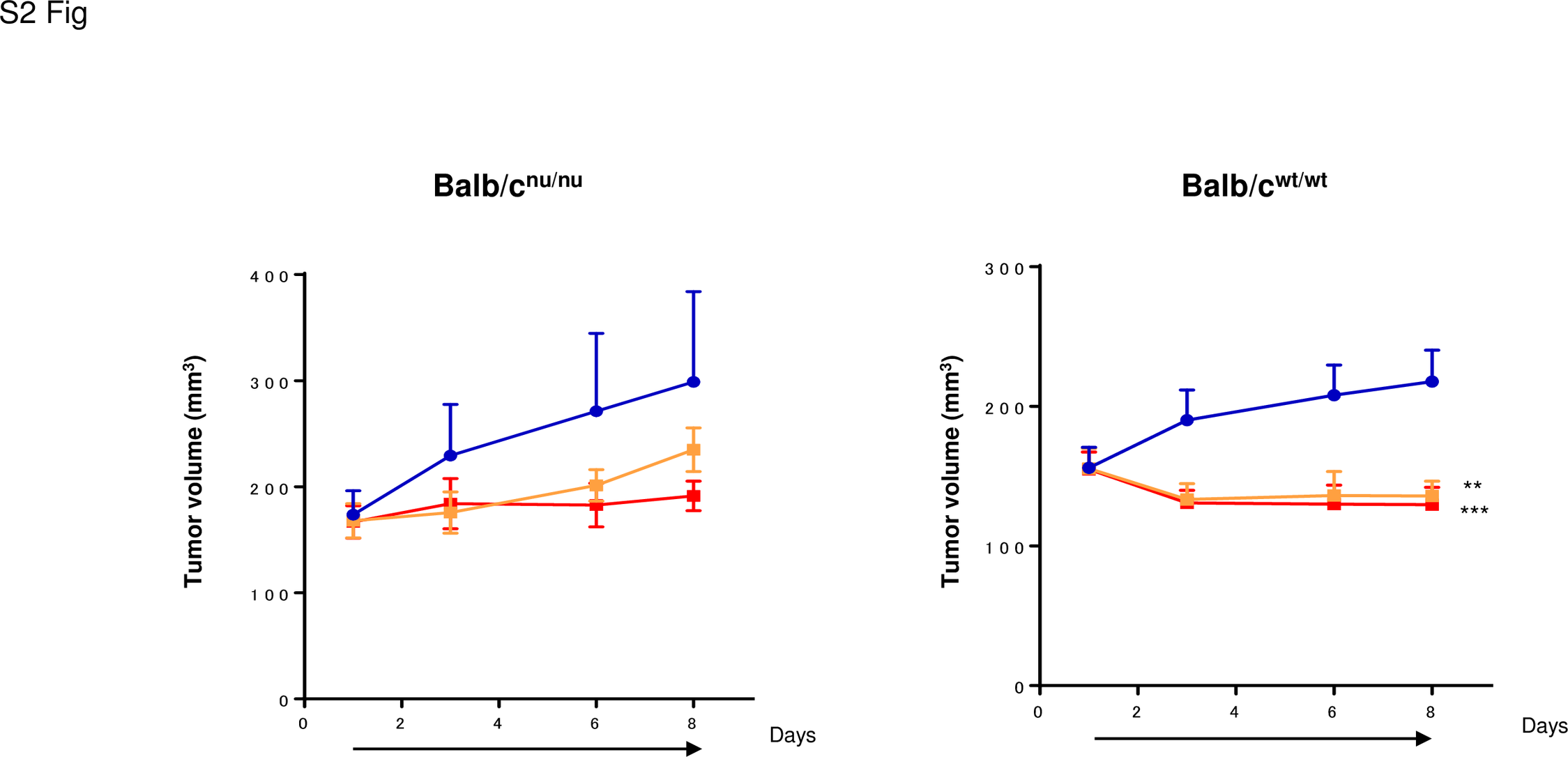

Supplement: S2 Fig — Immunodeficient mice (Balb/cnu/nu) and immunocompetent mice (Balb/cwt/wt) inoculated with BNL cells were randomized into groups of 5 with an average tumor volume size of 155 mm3 (Balb/cwt/wt mouse) or 170 mm3 (Balb/cnu/nu mouse) (Day 1) and then treated with vehicle (blue circle), 3 mg/kg lenvatinib (orange square), or 10 mg/kg lenvatinib (red square) once daily, indicated by the black arrow. Error bars represent the SEM. ****P<0.0001, Dunnett’s test vs. vehicle (n = 5). (TIF) [file pone.0212513.s002.tif]

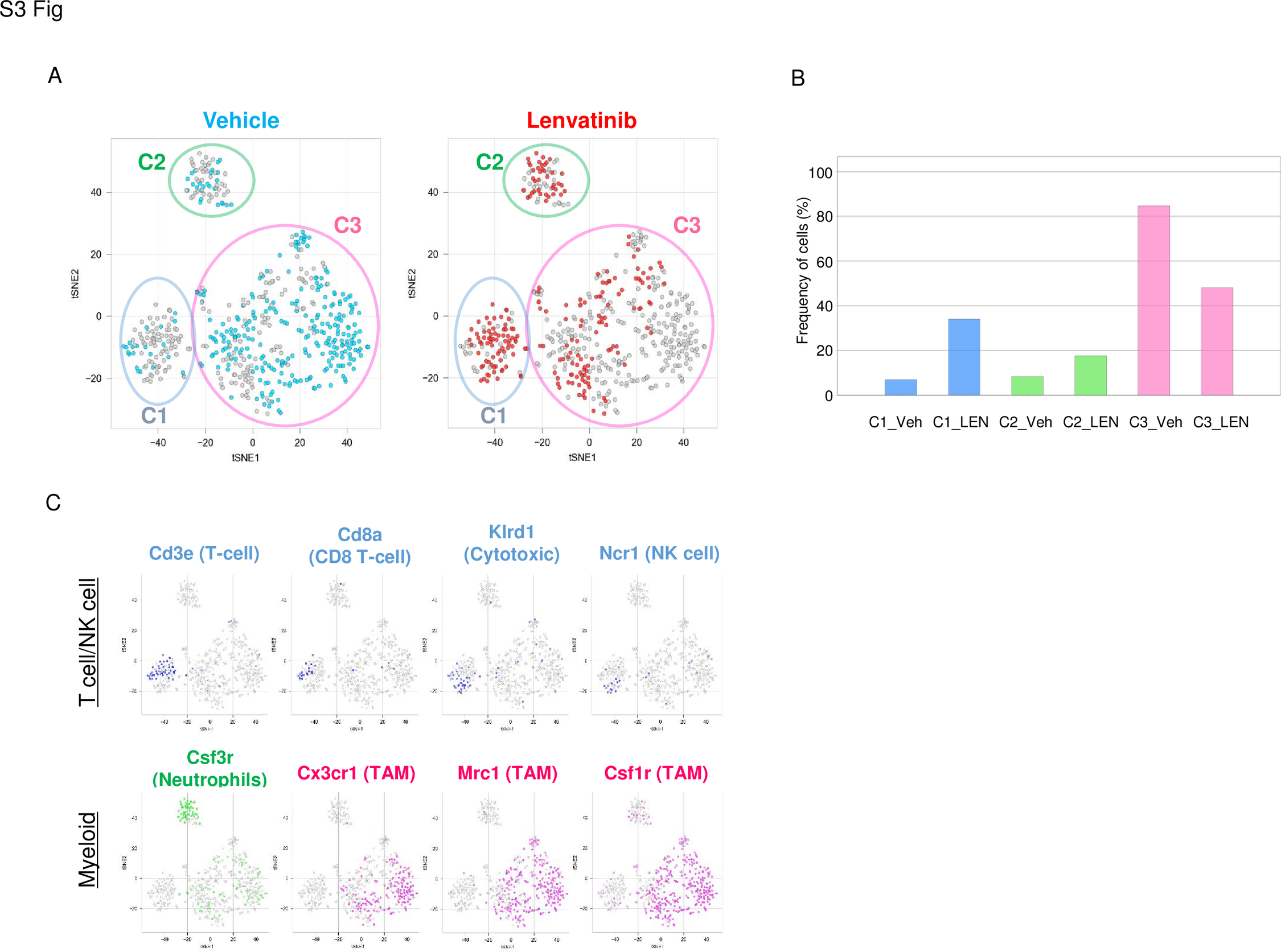

Supplement: S3 Fig — A. Single cells (521) are depicted in the tSNE two-dimensional space, where each plot represents each of the cells. Vehicle-treated cells are shown in cyan on the left; lenvatinib-treated cells are shown in red on the right. Cells were separated into three distinct clusters (C1: blue, C2: green, and C3: pink). B. Comparison of the changes in the proportions of cells in the three clusters between vehicle- and lenvatinib-treated cells. C. Expression levels of eight cell-specific marker genes. (TIF) [file pone.0212513.s003.tif]

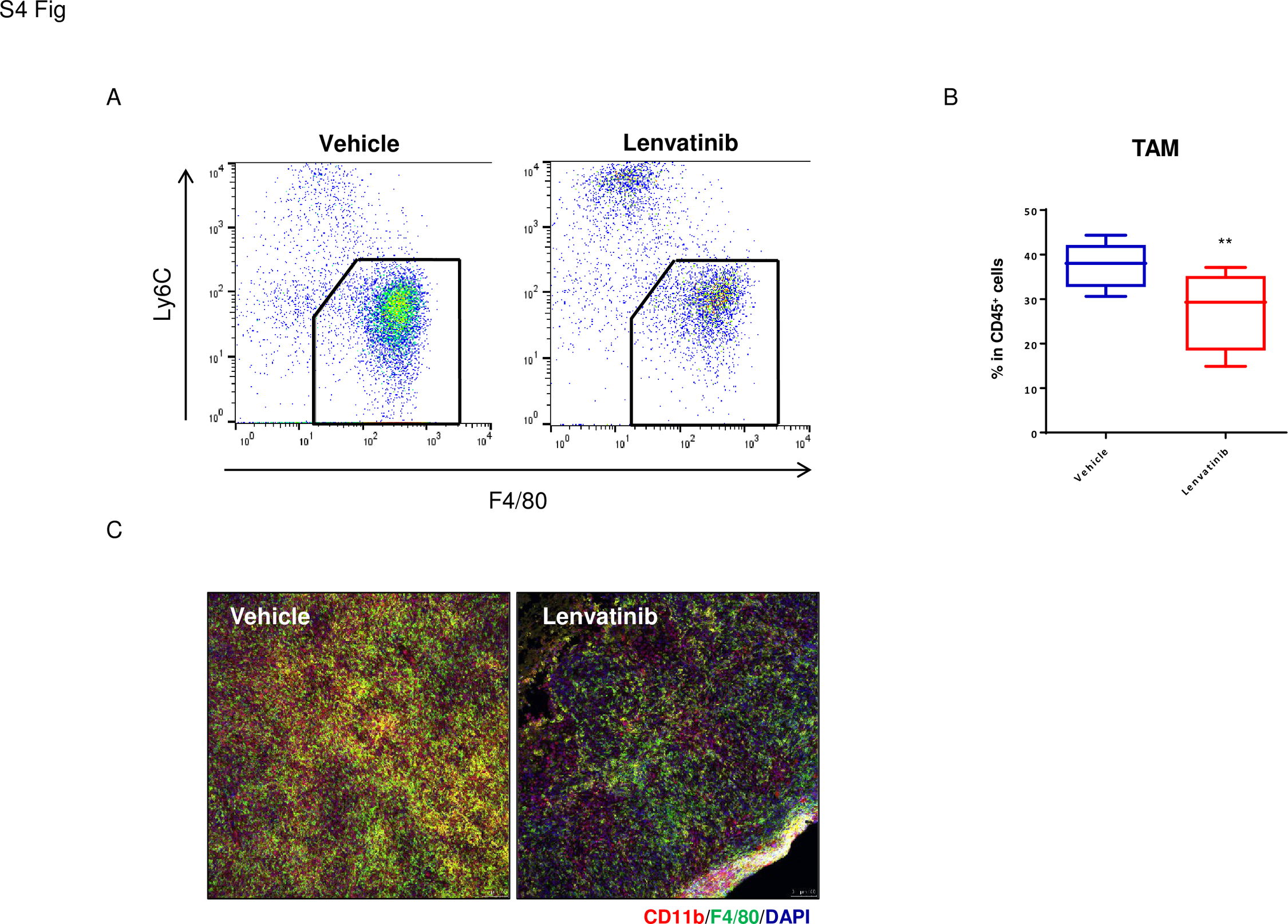

Supplement: S4 Fig — A and B. Representative profiles from flow cytometric analysis showing TAMs gated as a CD45+CD11b+Ly6G-Ly6C-F4/80+ population in tumors derived from vehicle- or 10 mg/kg lenvatinib-treated mice are shown. The percentage of the TAM population among the CD45+ cells is plotted. **P<0.01, unpaired t-test (n = 10). C. Representative images from immunohistochemical analyses showing CD11b (red) and F4/80 (green). TAMs are stained yellow. (TIF) [file pone.0212513.s004.tif]

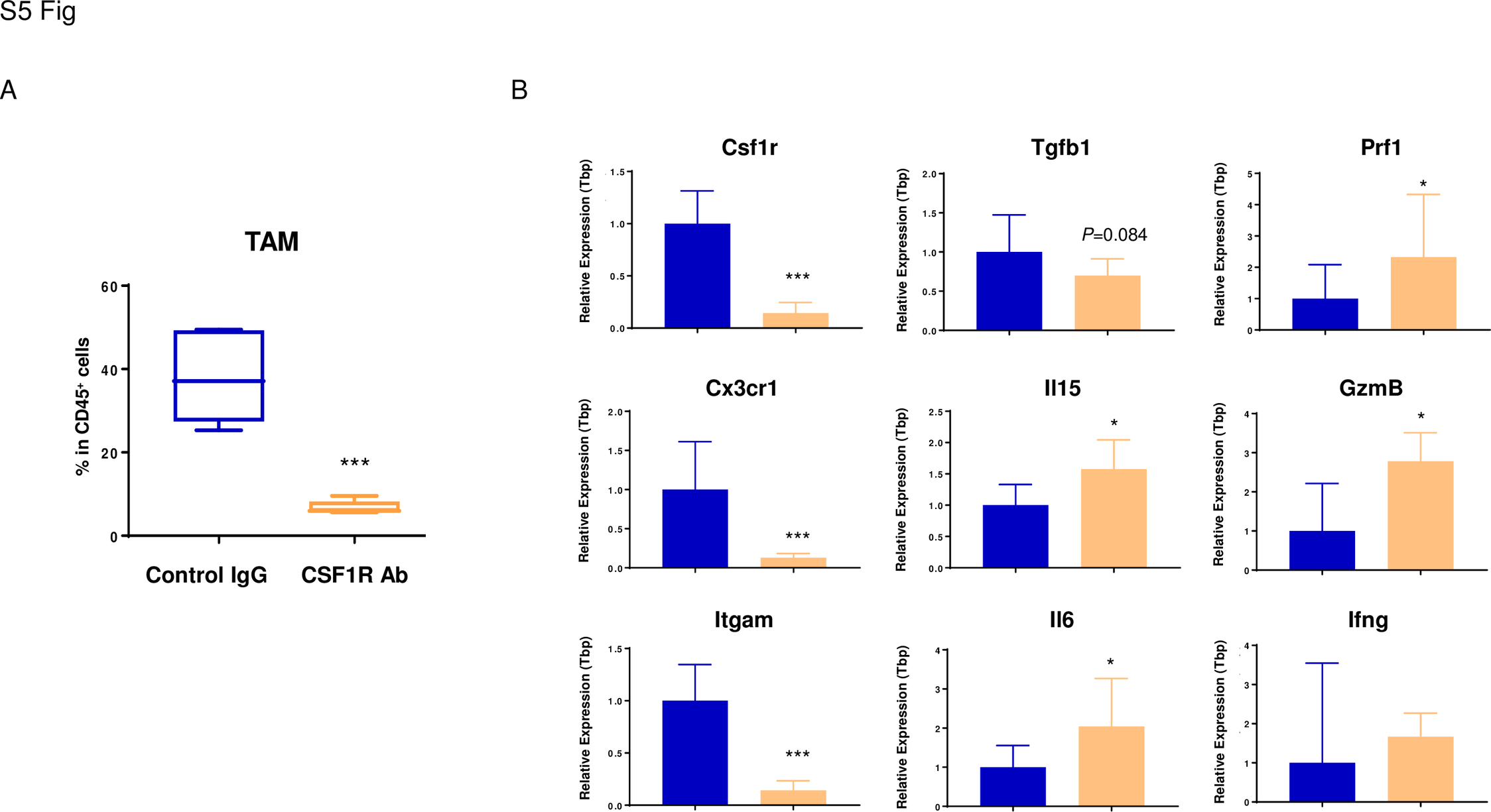

Supplement: S5 Fig — A. Tumor tissues were harvested on Day 8, and TAM depletion by the anti-CSF1R antibody was confirmed by flow cytometry. B. Gene expression profiles of Csf1r, Cx3cr1, Itgam, Tgfb1, Il15, Il6, Prf1, GzmB, and Ifng in tumor tissues from control IgG-treated mice (blue) and anti-CSF1R antibody-treated mice (orange) in quantitative PCR analysis. *P<0.05, ***P<0.001, unpaired t-test (n = 6). (TIF) [file pone.0212513.s005.tif]

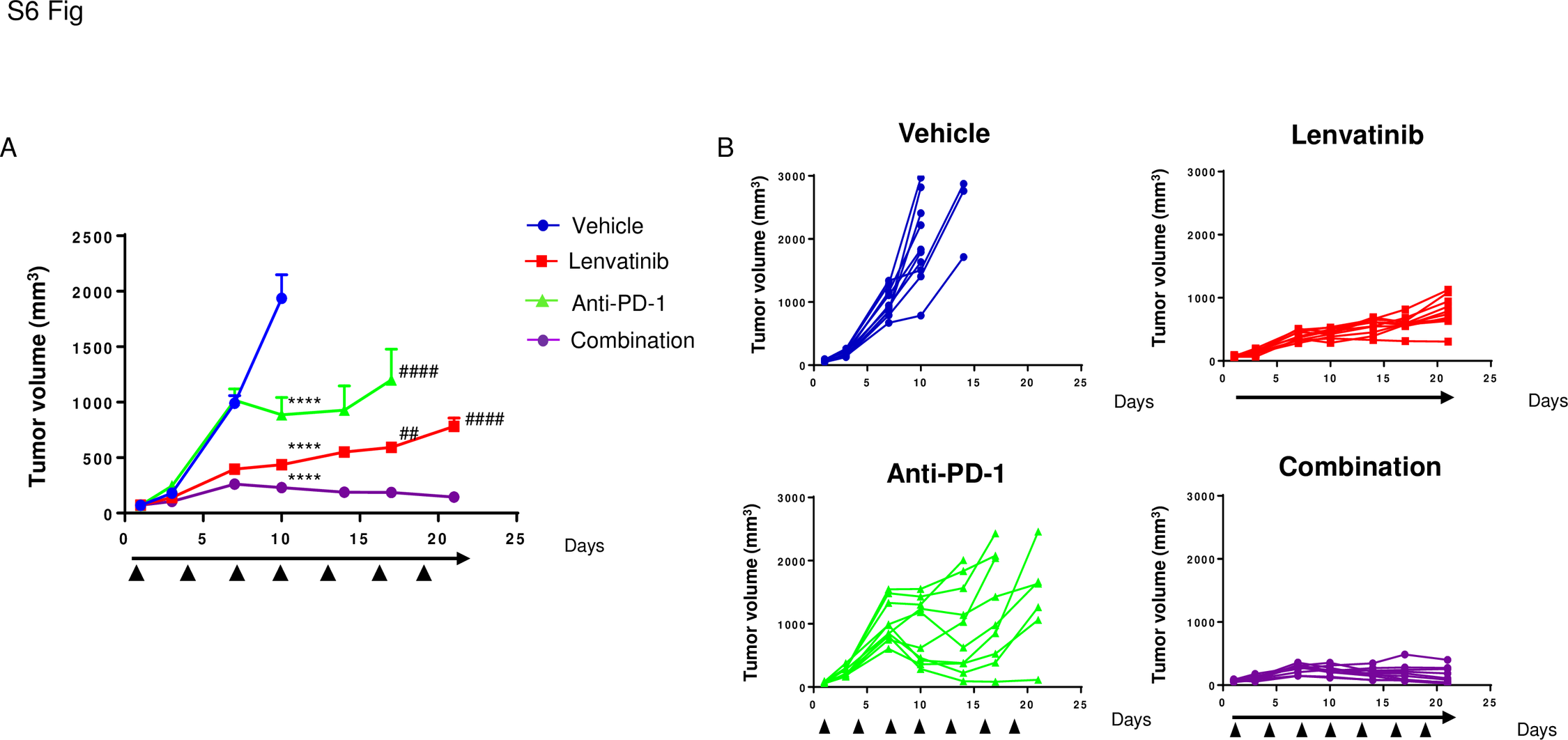

Supplement: S6 Fig — A. Mice were inoculated with B16-F10 cells and randomized into groups of 10 with an average tumor volume size of 71 mm3 (Day 1) and then treated with vehicle (blue circle), 10 mg/kg lenvatinib (red square) once daily, anti-PD-1 at 500 μg/mouse (green triangle) once every 3 days, or the combination (purple triangle). Lenvatinib treatment is indicated by the black arrow, and anti-PD-1 treatment is indicated as black triangles. B. Changes in tumor size for individual mice are shown for each group. *P<0.05, ****P<0.0001, Dunnett’s test vs. vehicle; ##P<0.01, ####P<0.0001 vs. combination. (TIF) [file pone.0212513.s006.tif]

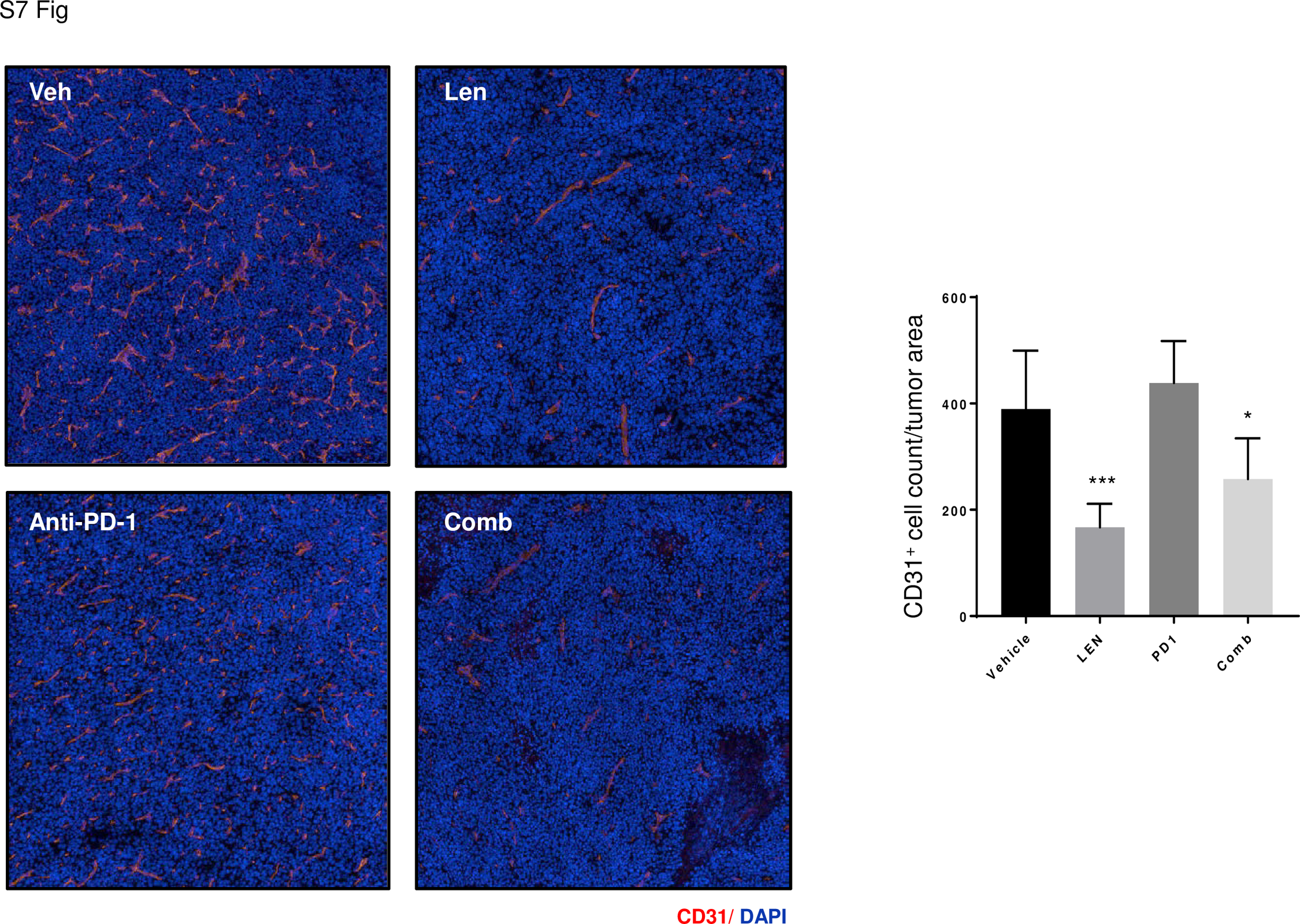

Supplement: S7 Fig — A. Representative images from immunohistochemical analysis showing CD31 (red) and DAPI (blue). B. The number of CD31-stained cells and the area of the tumor tissue slice were determined for each mouse by using HALO; the CD31+ cell number was then divided by the tumor tissue area (mm2) and plotted. *P<0.05, ***P<0.001, Dunnet’s test vs. vehicle. (TIF) [file pone.0212513.s007.tif]

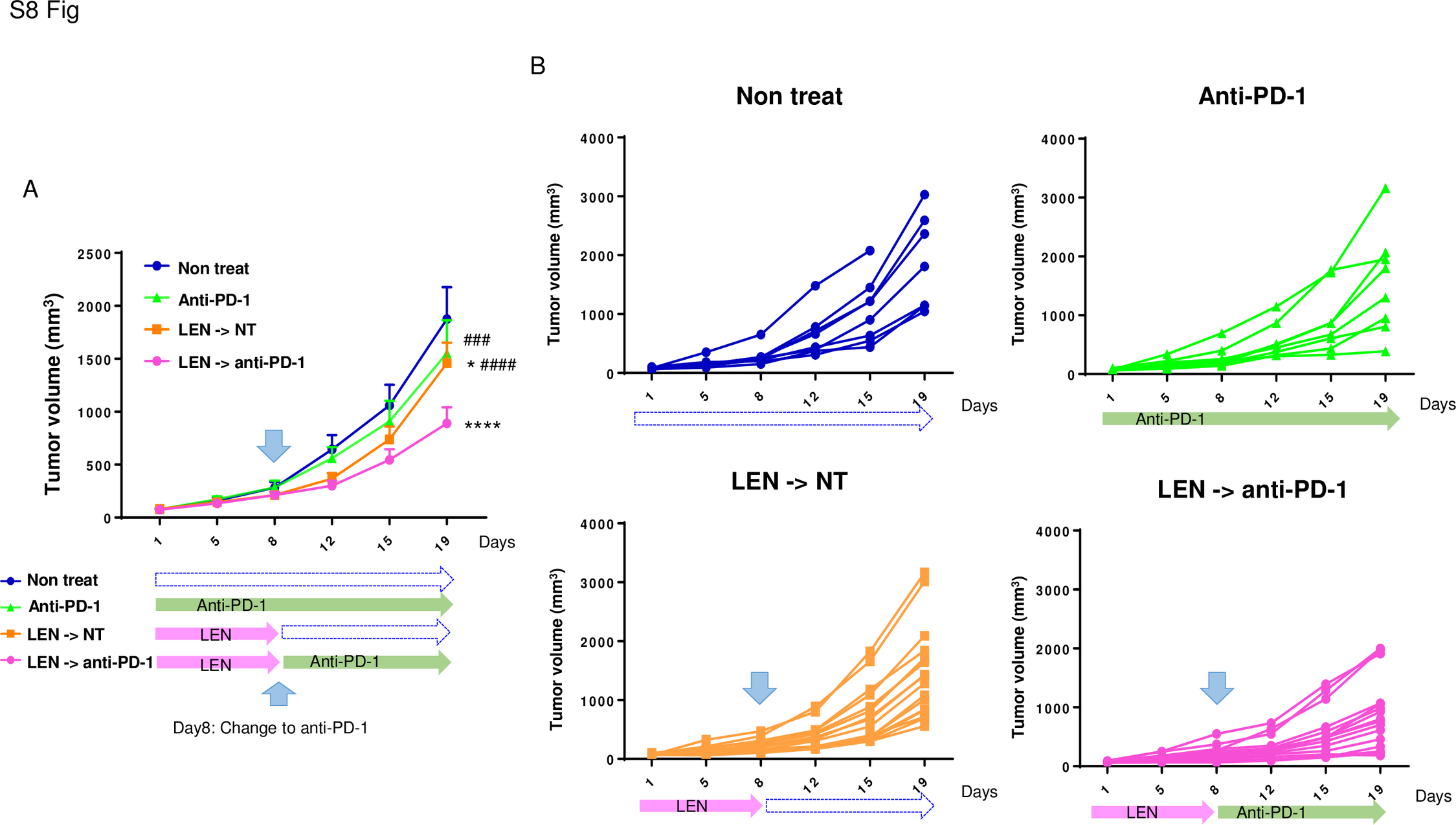

Supplement: S8 Fig — A. Mice were inoculated with CT26 cells and randomized into 3 groups with an average tumor volume of 78.4 mm3 (Day 1) as follows: nontreatment, 8 mice; anti-PD-1 treatment, 8 mice; and lenvatinib treatment, 32 mice. Mice were then treated with nontreatment (blue circle) or anti-PD-1 at 200 μg/mouse (green triangle) once every 3 days. The lenvatinib-treated mice were further randomized into 2 groups of 16 mice on Day 8 at an average tumor volume of 212 mm3 and then treated with anti-PD-1 at 200 μg/mouse (pink circle) once every 3 days or nontreatment (orange square). Lenvatinib treatment is indicated by the pink arrow and anti-PD-1 treatment is indicated by the green arrow. Nontreatment is indicated by the blue-framed arrow. B. Changes in tumor size for individual mice are shown for each treatment group. Error bars represent the SEM. *P<0.05, ****P<0.0001, Dunnett’s test vs. vehicle; ###P<0.001, ####P<0.0001, vs. combination. (TIF) [file pone.0212513.s008.tif]

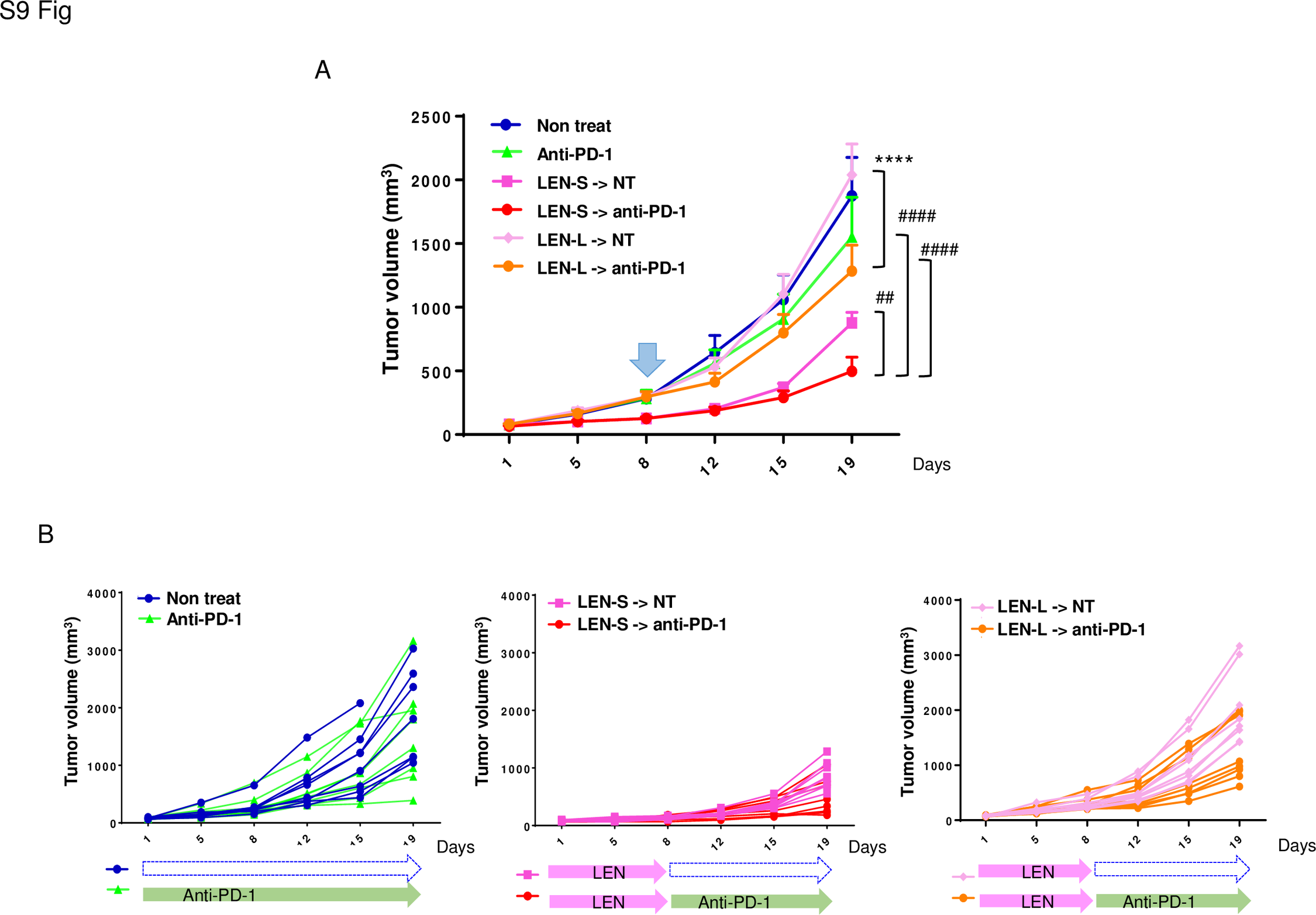

Supplement: S9 Fig — Using the same data as those used in the legend to S8 Fig, lenvatinib-treated mice were further divided into two groups based on the tumor volume on Day 8: the lenvatinib-sensitive relatively small tumor group (LEN-S; mean TV, 127 mm3; n = 16) and the lenvatinib moderate sensitive relatively large tumor group (LEN-L; mean TV, 296 mm3; n = 16). Tumor growth was then re-analyzed according to anti-PD-1 treatment (n = 8) or nontreatment (n = 8) for both the LEN-S and LEN-L group. Tumor growth in mice treated with nontreatment (blue circle), anti-PD-1 (green), LEN-S followed by anti-PD-1 (red) or nontreatment (dark pink), and LEN-L followed by anti-PD-1 (orange) or nontreatment (pink). B. Changes in tumor volumes for individual mice are shown for each treatment group. Error bars represent the SEM. ****P<0.0001, ****P<0.0001, Dunnett’s test vs. LEN-L followed by anti-PD-1; ##P<0.01, ###P<0.001, ####P<0.0001, vs. LEN-S followed by anti-PD-1. (TIF) [file pone.0212513.s009.tif]

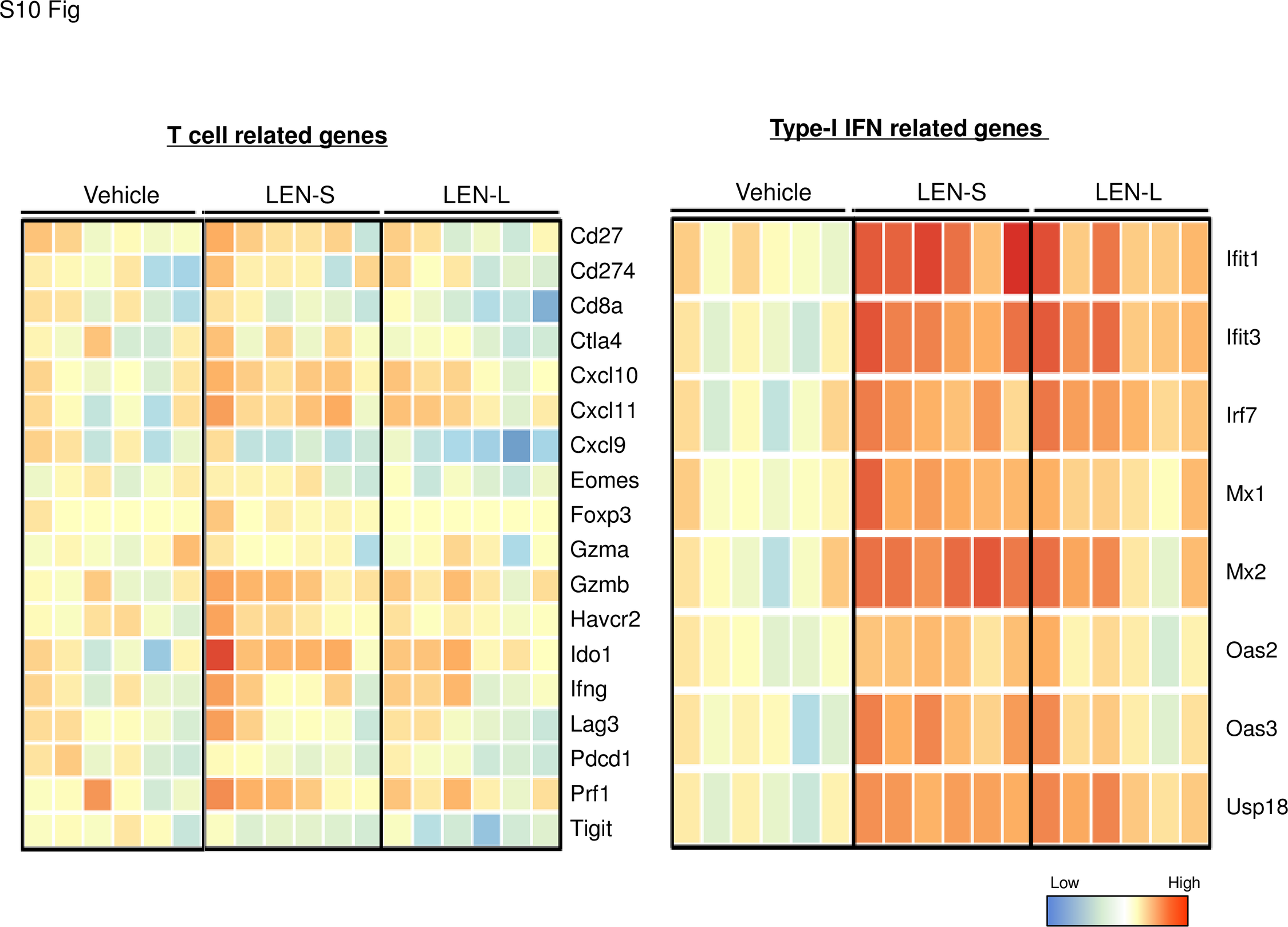

Supplement: S10 Fig — Using RNA-Seq data from the LEN-S and LEN-L tumors, gene expression levels of T cell-related genes and type-I IFN-related genes compared with vehicle were plotted as heatmaps. The gene expression level indicator is shown at the bottom right. (TIF) [file pone.0212513.s010.tif]

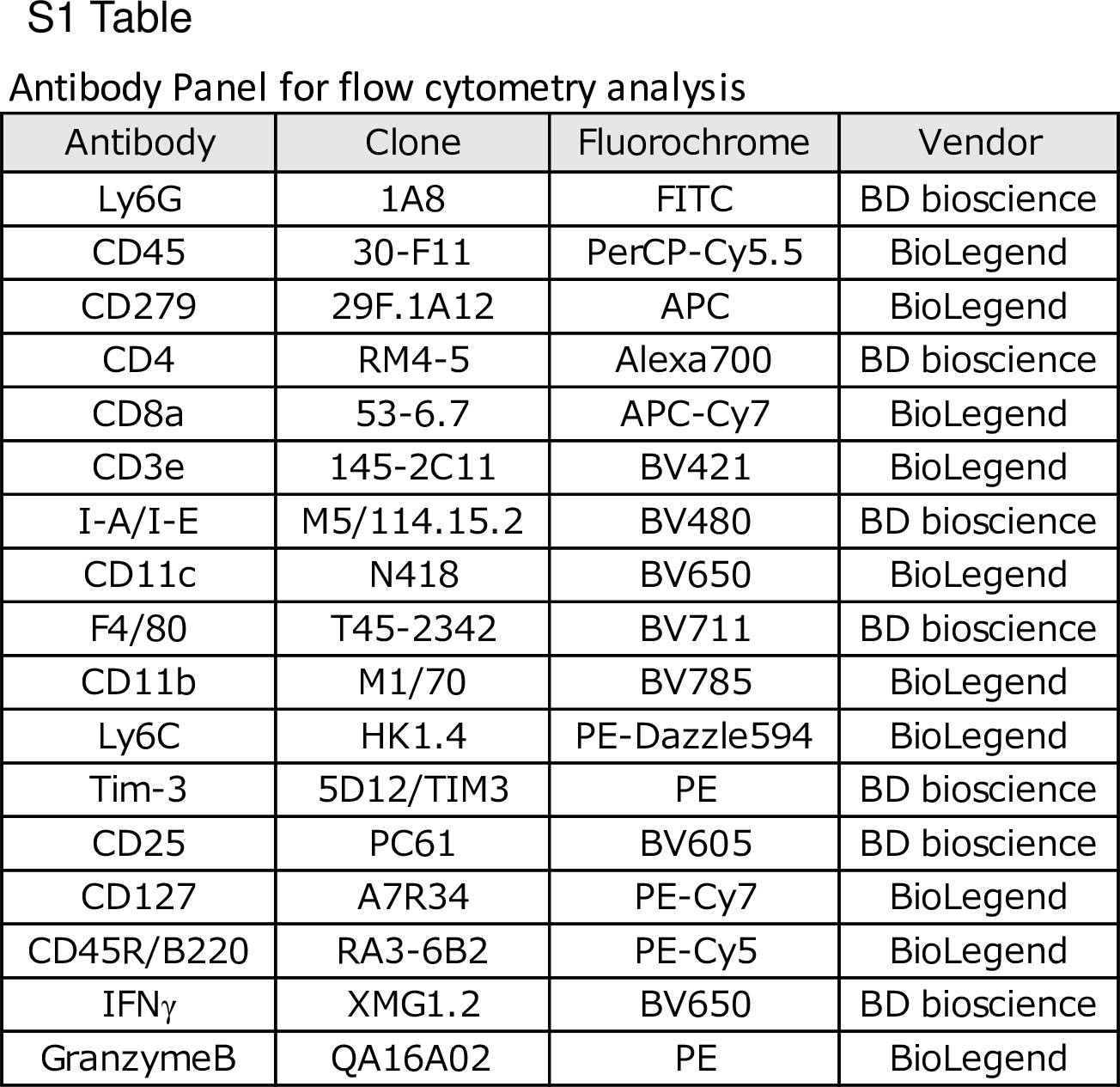

Supplement: S1 Table — (TIF) [file pone.0212513.s011.tif]

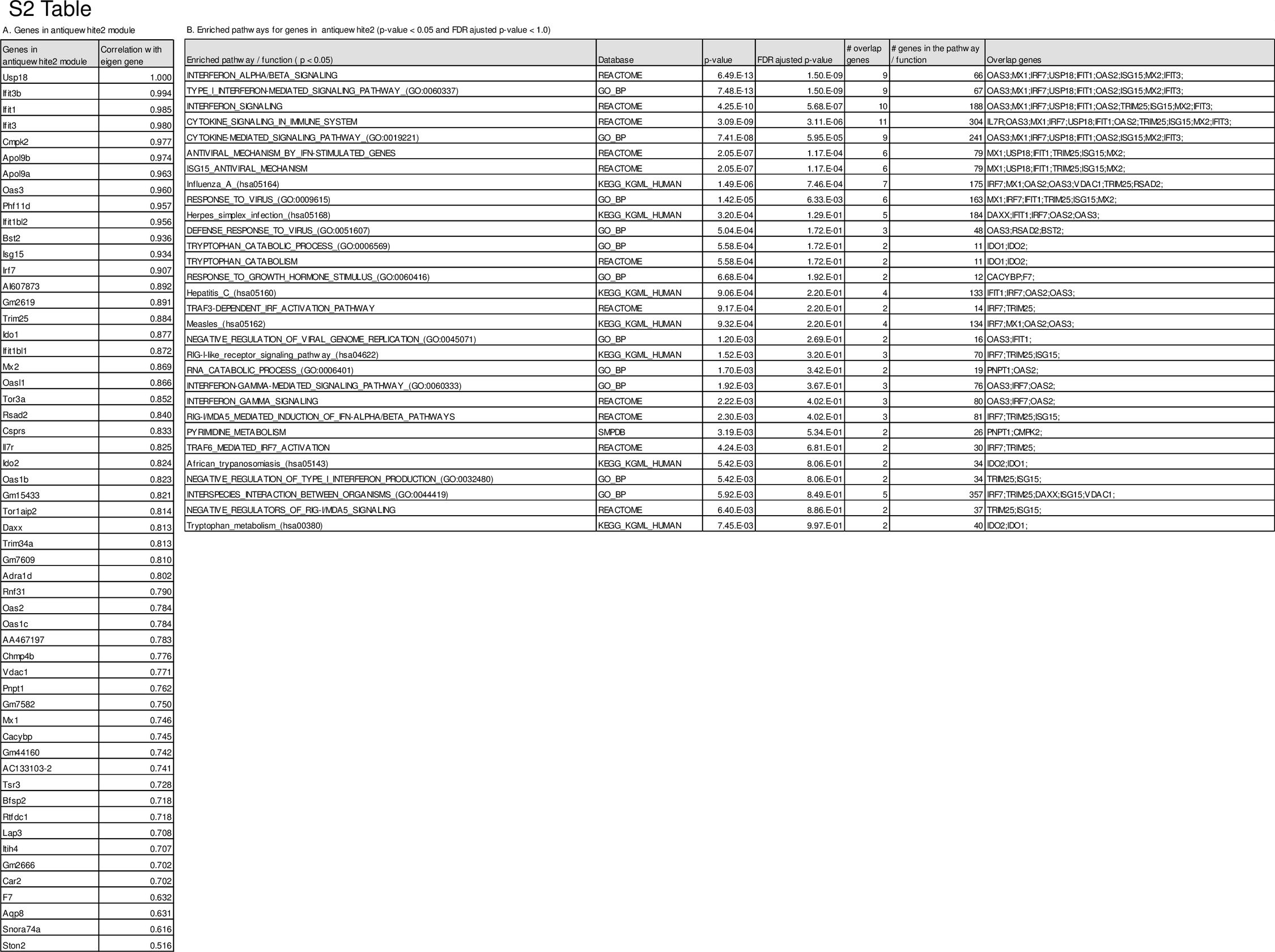

Supplement: S2 Table — The module genes and their correlation with the module eigengene and enriched biological pathways. (TIF) [file pone.0212513.s012.tif]

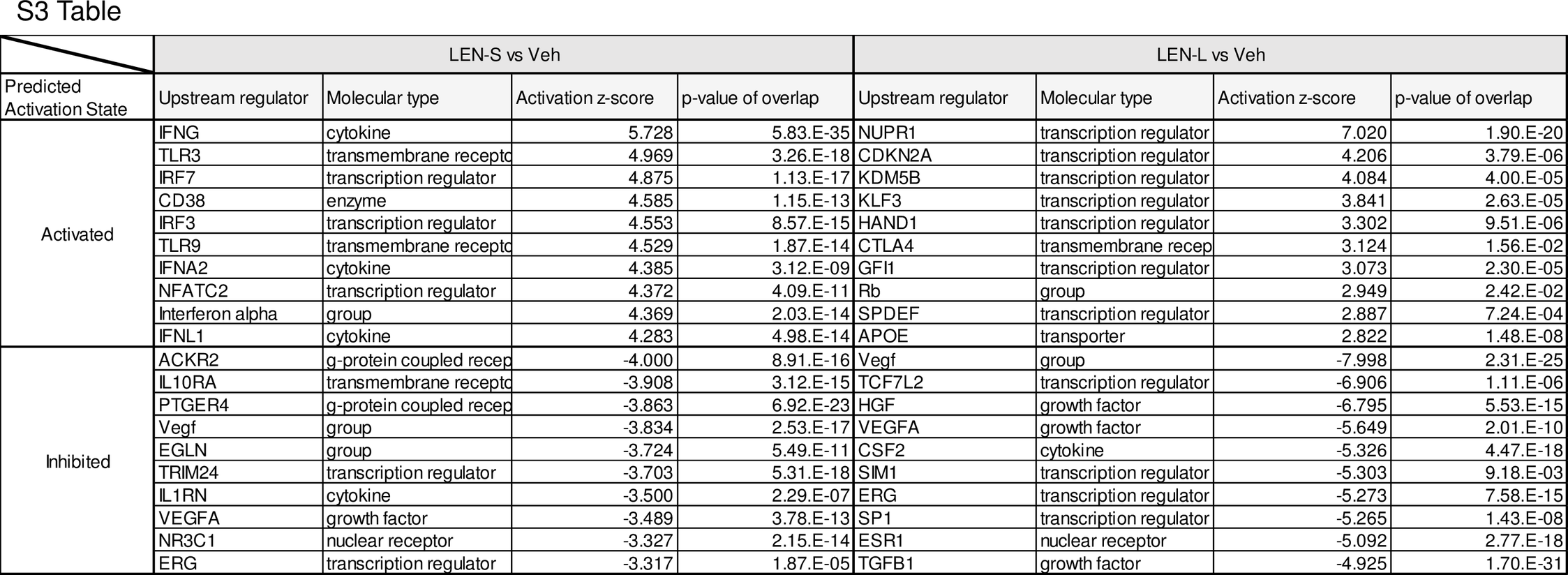

Supplement: S3 Table — The top ten activated or inhibited upstream regulators in LEN-S and LEN-L compared with vehicle are listed. (TIF) [file pone.0212513.s013.tif]
